# Behavioral characterization of dynamic facial expression perception in rhesus monkeys *(Macaca mulatta)* using naturalistic and synthetic stimuli

**DOI:** 10.1007/s10071-026-02109-6

**Published:** 2026-10-05

**Authors:** Ramona Siebert, Nick Taubert, Martin A. Giese, Peter Thier

**Affiliations:** 1 Cognitive Neurology Laboratory, Hertie Institute for Clinical Brain Research, University of Tübingen, Tübingen, Germany; 2 Computational Sensomotorics, Centre for Integrative Neuroscience & Hertie Institute for Clinical Brain Research, University of Tübingen, Tübingen, Germany; 3 Graduate School of Neural and Behavioural Sciences, University of Tübingen, Tübingen, Germany

**Keywords:** Facial expression, Rhesus macaque, Social cognition, Face avatar

## Abstract

Facial displays are an important channel of communication for primates, yet it remains unclear based on which criteria monkeys evaluate facial expressions. We trained two rhesus macaques to categorize videos of four facial expression types (neutral, lip-smacking, silent bared-teeth (SBT) and open-mouth threat displays) and then tested generalization to novel individuals and avatars while measuring pupillary responses. The animals were able to categorize facial expressions and generalized their classifications to novel, untrained videos, although performance was not perfect. Misclassifications were not driven solely by visual similarity between expression categories, as demonstrated by a novel automated approach integrating deep learning-based motion tracking with the Macaque Facial Action Coding System to enable objective quantification of facial action units. Instead, open-mouth threats were readily categorized correctly and distinguished from lip-smacking, while also eliciting the strongest arousal responses. By contrast, categorization of SBT displays varied substantially across stimulus identities, influenced by body weight, gaze direction, and coordinated movements of eyebrows and ears. Morphed avatar expressions were categorized according to expression component intensity, demonstrating graded perception. Avatar manipulations revealed that categorization was robust against lack of coherent motion and lack of facial realism beyond a basic level of texture. However, human facial expressions elicited no systematic categorizations or differential arousal. Our findings indicate that conspecific facial expressions were perceived as functionally meaningful, context-dependent social signals shaped by all components of facial behavior and signaler characteristics, rather than fixed, mouth movement-defined categories.

**Supplementary Information:**

The online version contains supplementary material available at https://doi.org/10.1007/s10071-026-02109-6.

## Introduction

The ability to distinguish and interpret facial expressions is an important social skill for all primate species. Since Darwin (1872) pioneered the systematic investigation of facial displays in humans and animals, many ethological studies in non-human primates have provided descriptions of facial movements and the situations in which they usually occur (e.g. Andrew 1963; Hinde 1966; Maestripieri 1997; Partan 2002; Preuschoft 2000; van Hooff 1967). Laboratory studies have reported how macaque monkeys visually scan facials displays (Gothard et al. 2004; Mosher et al. 2011; Nahm et al. 1997; Siebert et al. 2020) and examined neural responses to viewing conspecific facial expressions (e.g. Gothard et al. 2007; Hadj-Bouziane et al. 2008; Hasselmo et al. 1989; Hoffman et al. 2007; Taubert et al. 2020; Yang and Freiwald 2021; Zhu et al. 2013). The most frequently employed expressions in these studies were aggressive open-mouth threat, affiliative/appeasing lip-smacking with puckered lips and rapid opening and closing of the mouth, and fearful/submissive fear-grin or -grimace, more objectively called the silent bared-teeth display (SBT), with mouth corners and lips retracted to expose the teeth. However, despite the widespread use of these stereotypical categories, few studies have explicitly asked monkeys to behaviorally discriminate facial expressions. Kanazawa (1996) reported that one Japanese macaque differentiated facial displays in a match-to-sample paradigm along a “subordinate – dominant” dimension, distinguishing SBT and threat faces by mouth shape and eye width, and a “neutral – tense” dimension, separating relaxed faces from faces with raised eyebrows, whereas the macaque only distinguished the human smile among other human expressions. This study only used one Japanese macaque’s and one human’s displays as stimuli, leaving unclear whether the subject resorted to any semantic information about expression categories or simply differentiated dissimilar pictures. Parr and Heintz (2009) performed a similar experiment with seven rhesus macaques and the categories open-mouth threat, SBT, play face and scream face. The macaques struggled when the identity of the sample, the matching and the non-matching stimulus were all different and confusion apparently occurred due to shared features between expression categories, especially mouth opening and teeth exposure. However, overlapping features alone could not explain the overall error pattern. Notably, the macaques exhibited a bias for selecting the play face, presumably the most positively valued among the offered categories. An analogous approach was used by Micheletta et al. (2015) who tested three crested macaques and reported that the visual similarity between expressions was not correlated with performance, and speculated that functional similarity between expressions could be responsible for errors. Taubert and Japee (2024) trained four rhesus macaques to select a “positive” facial expression (neutral or lip-smack) over a “negative” expression (threat or SBT). When tested with novel images, the macaques most frequently selected the lip-smack stimuli and least frequently selected the threat stimuli, as expected, but also selected two SBT displays over most neutral faces, while the other three SBTs ranked among the threat displays, suggesting variable valence of SBTs. All four studies relied on static images, although Micheletta et al. (2015) also included dynamic video stimuli and anecdotally noted improved performance. A more nuanced study was conducted in chimpanzees: using a chimp avatar with individually movable action units, Parr et al. (2008) showed that facial expressions were discriminated by key features along two dimensions, the degree of mouth opening and lip posture (puckered or retracted).

While these studies have demonstrated that macaques are able to distinguish facial expressions in experimental settings, it remains unclear (I) which strategy they used to discriminate expressions and (II) which facial motion, shape and appearance features are necessary and sufficient for the perception and differentiation of a facial expression category. To answer these questions, we trained two rhesus macaques to explicitly discriminate videos of neutral, lip-smacking, SBT and threat displays in a forced-choice categorization paradigm, avoiding a potential bias to select more pleasant expressions in a match-to-sample configuration. We subsequently assessed them using novel expression videos in catch trials and measured physiological arousal through pupillary responses. For part I), the test videos were filmed real macaques and a naturalistic monkey avatar. We reasoned that if the macaques used elementary facial features to make their decisions without attaching any meaning to the videos, they would perform comparably well across expression categories for the new test videos, with mistakes occurring among visually similar expressions and without physiological reactions to the videos. However, if the macaques perceived the videos as the described mouth movement-defined morphological categories (neutral, lip-smacking, SBT and open-mouth threat), each with discrete meaning, they would exhibit the same performance and error pattern, but experience differential arousal when seeing the different expression categories. To quantify similarity among the expressions, we combined the Facial Action Coding System (FACS), which allows the objective, muscle-based description of facial movements (Ekman and Friesen 1978; Ekman et al. 2002; Parr et al. 2010) and deep learning-based motion tracking (DeepLabCut) (Mathis et al. 2018; Nath et al. 2019). If the meaning the macaques extracted from the stimuli is not fully captured by the defined categories, inconsistent performance across expression categories and test identities should occur and visual similarity should fail to explain the pattern of miscategorizations. Instead confusions between expressions with similar function should occur, like lip-smacking and neutral, lip-smacking and SBT or SBT and threat, and a clear distinction between lip-smacking and threat. However, if they only learned the association of the rewarded target for each individual training video, they should fail to categorize the new videos according to expression type, although rhesus macaques were able to categorize action videos in a similar paradigm (Cui et al. 2023). For part II), we made use of a monkey avatar to continuously morph one expression into another, to remove motion coherence, to systematically change the facial appearance by gradually decreasing realism (no fur, no color, no texture, no shape), to change the species-specific facial form to a human avatar and to change the species-specific motion pattern to human expressions. Note that we use the conventional term facial expression interchangeably with facial display, facial configuration, facial behavior or facial movement pattern, without assuming any expression of an emotional state.

## Materials and methods

### Subjects

Data was collected from two adult male rhesus macaques (*Macaca mulatta*), monkey 1 (7 years, 10.5 kg) and monkey 2 (8 years, 8.5 kg). They were born at the German Primate Center, raised in social groups, and pair-housed in our laboratory with visual and auditory contact to conspecifics. For both monkeys, this was the first scientific experiment in which they participated. Following chair training, they were implanted with individually adapted titanium head posts for head immobilization during eye tracking and subsequently planned neurophysiological experiments. All surgical procedures were conducted aseptically under general anesthesia and control of vital parameters. After recovery, the monkeys were gradually trained to accept the head immobilization and to receive liquid rewards (juice and water) for fixating dots and images on a computer screen. All animal procedures were approved by the veterinary administration (Regierungspräsidium Tübingen, Abteilung Tierschutz) and complied with German law (Tierschutz-Versuchstierverordnung) and the National Institutes of Health’s Guide for the Care and Use of Laboratory Animals.

### Visual stimuli

**Real monkeys**: Training stimuli consisted of 13 videos per expression category from diverse sources: 13 rhesus macaques from our and neighboring laboratories (known and unknown to subjects) sitting in a primate chair with their heads free to move, natural environment footage (www.youtube.com/@rhesusvideo6831, channel photo credit: Lauren Brent), and still photographs (PrimFace database: http://visiome.neuroinf.jp/primface/, funded by Grant-in-Aid for Scientific research on Innovative Areas, “Face Perception and Recognition” from the Ministry of Education, Culture, Sports, Science, and Technology (MEXT), Japan). This provided variability in background, face size, and dynamic content. The test stimuli (all male rhesus macaques) comprised one set of expression videos of monkeys contained in the training set (unknown individuals) which had not been shown during training, and nine new identities. Eight of these (ID 1–2, 5–9) were monkeys from neighboring laboratories (ages 12–17, weight 8.8–15.6 kg), unknown to the subjects, and one corresponded to the subject’s cage partner (ID 3 and 4, respectively). Four test expression sets (ID 1–4) comprised the complete desired expression repertoire (neutral, lip-smacking, silent bared-teeth and open-mouth threat), whereas five individuals (ID 5–9) did not show the silent bared-teeth display. Facial behaviors were elicited by the experimenter interacting with the monkey, mimicking macaque displays and showing positive (treats) and negative (tools) stimuli. Each test monkey was filmed in a single recording session under standardized conditions seated in a primate chair with unrestrained head against a neutral white background. Test videos were cropped to show the heads only, cut into clips of single continuous facial displays, manually scored according to the Macaque Facial Action Coding System (MaqFACS) (Parr et al. 2010) and assigned to four expression categories. “Neutral”: no activation of mouth action units (AUs) and little to no eyebrow or ear movements. “Lip-smacking”: AU18i (true lip pucker) and AD181 (lip-smacking action descriptor). “Silent bared-teeth” (SBT) display (fear-grin): AU10 (upper lip raiser), AU12 (lip corner puller), AU16 (lower lip depressor), AU25 (lips parted) and optionally AU26 (jaw drop). “Open-mouth threat”: AU25 (lips parted) and either AU26 (jaw drop) (without any AU12) or AU27 (mouth stretch). Only clear expressions were used. Similar definitions for macaque facial expression categories were employed by others (Parr et al. 2010; Taubert and Japee 2024).

**Monkey and human avatars**: The monkey avatar was created from an MRI-based mesh surface controlled by ribbons modeled after rhesus macaque facial muscles, driven by motion-captured facial movements (Siebert et al. 2020; Taubert et al. 2021). We have previously demonstrated that this monkey avatar elicits behavioral reactions similar to real macaque videos and does not fall into the “uncanny valley” of aversion in the visual perception of rhesus macaques towards synthetic agents (Siebert et al. 2020). The human avatar used equivalent control points following human muscle anatomy. In addition to the pure, prototypical motion-captured expressions, the monkey avatar displayed expressions morphed in 25% intensity steps between prototypical category pairs. Additional test conditions included variations of the prototypical expressions monkey avatar: temporally scrambled frames, point-light displays (movement patterns without texture) and degraded realism versions (furless, grayscale, wireframe); as well as human expressions (smiling/happy, gasping/fearful, scowling/angry) on human and monkey avatars. More details on the avatar stimuli are available in the Supplementary Methods.

### Experimental paradigm and procedure

**Fig. 1:**
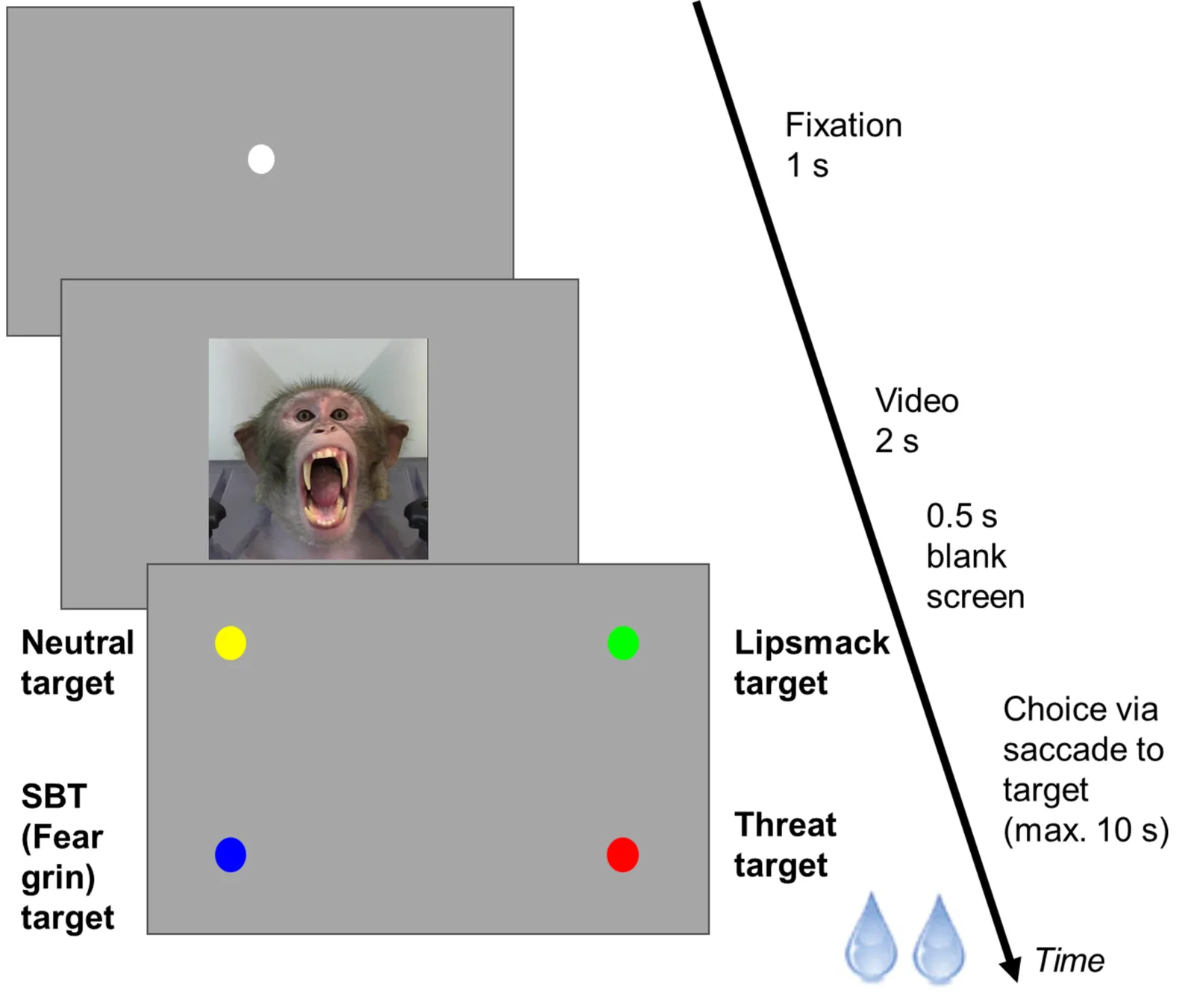
Forced-choice expression categorization paradigm

Subjects sat head-restrained in a primate chair 60 cm in front of a computer monitor in a darkened booth. Stimuli were presented via the NREC open-source control system (https://nrec.neurologie.uni-tuebingen.de/) on a gray background. Eye position and pupil size (vertical diameter) were recorded with an eye tracker (ISCAN ETL-200, 120 Hz). A trial was initiated by fixation on a dot (radius 0.2° visual angle) for 1 s, followed by a 1–2 s centrally presented video (12° x 12°) and 0.5 s of blank screen. Then four differently colored targets (0.4° radius) appeared in the screen corners (each 12° horizontally and 7° vertically from the screen center). Subjects indicated the perceived expression category via a saccade to the corresponding target (detection window 6° x 6°) (Fig. 1). Target-category associations were learned through reward contingencies assigned by the experimenter. Eye positions outside the bounds of the video or failure to saccade to any of the targets after 10 s aborted the trial. During the training phase, subjects were only rewarded for choosing the correct target. Task training started with expression categories separately in blocks with a single target and a subset of stimuli, then successively more stimuli and more targets were added and more categories were nested until full randomization of trials was reached. The training procedure was flexibly adapted to the animal’s current needs and abilities, e.g. more intense training of videos that were frequently miscategorized. The entire training period, from the first time the animal sat inside the experimental booth to the start of the test phase, lasted a little over one year. Once performance stably exceeded 70% per category (Supplementary Fig. S1A-B), the test phase was started with new videos presented interleaved with the trained videos in 5–10% catch trials, in which choice of any target was rewarded. The performance on the trained videos throughout the test phase is shown in Supplementary Fig. S1C-D. In part I of the test phase, all new real macaque videos and the monkey avatar with prototypical expressions were tested. Then, the subjects were specifically re-trained on the prototypical expression avatar videos for approximately two weeks, which were subsequently incorporated into the training set. In part II, the morphed avatar expressions, the scrambled-frame avatar, the point-light avatar, the degraded realism avatars and the human avatar were tested. The morphed test stimuli were presented in 9.9% of avatars trials (among the prototypical avatar expressions), nested among the original training videos, i.e. in 7.7% of all trials. Testing sessions were conducted in blocks of 234 to 344 trials (depending of the percentage of catch-trials), with breaks between blocks, and lasted for as long as the animal was willing to work. When the animal stopped working before completion of a block, the remainder of the block was finished in the next testing session. Typically, monkey 1 completed between 1000 and 1500 trials per day and monkey 2 completed between 300 and 900 trials per day. We aimed to collect at least 50 trials per individual test video from each subject. Unfortunately, data collection for part II from monkey 2 could not be completed due to persistent administrative delays in the renewal of our animal testing license. Thus, only 18 (morphed avatar expressions), 30 (scrambled-frame avatar), 29 (point-light avatar), 15 (degraded realism avatars) and 29 (human avatar) repetitions per test video are available from monkey 2. The human facial expressions, displayed by both the human avatar and the monkey avatar, could only be tested with monkey 1 for the same reason.

### Quantification of facial movements

**Manual FACS coding**: MaqFACS AU activations per video were coded before data collection by R. Siebert, who is a certified MaqFACS coder.

**Fig. 2:**
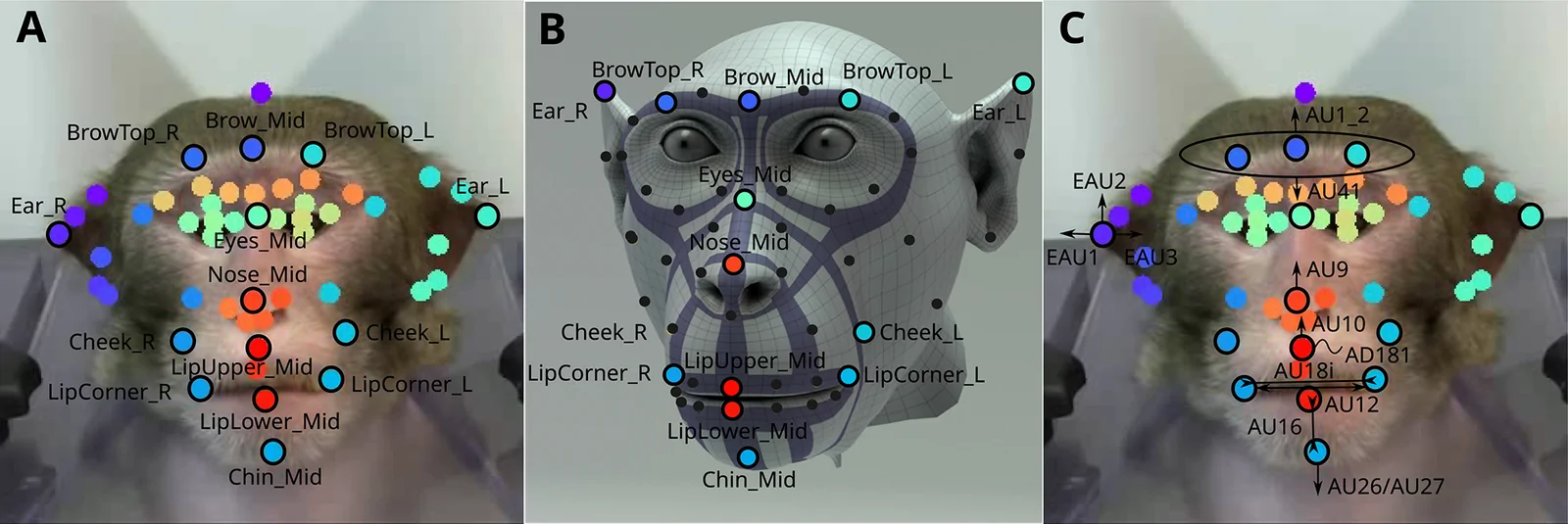
**(A)** Facial movements in the real monkey videos were tracked using DeepLabCut (pre-trained “primate face” model). Markers used to approximate MaqFACS AUs are outlined in black and labeled. **(B)** Avatar mesh joints. Joints equivalent to the employed DLC markers are color-coded and labeled accordingly. **(C)** Schematic of the automatic MaqFACS AU activation and intensity calculation, for details see Supplementary Table S1

**Automatic FACS coding**: Facial movements in the real macaque test videos were tracked via markerless pose estimation implemented in DeepLabCut (DLC) (Mathis et al. 2018; Nath et al. 2019), using the pre-trained “primate face” from the DeepLabCut Animal Zoo, provided by Claire Witham, Centre for Macaques, MRC Harwell, UK (Fig. 2A) and a model confidence threshold of 0.6. The Eyes_Mid marker was used as a reference and its position was subtracted from all other markers in order to obtain local motion only. For the avatars, mesh joints equivalent to the DLC markers were used (Fig. 2B). AU activations were coded based on the movements of the marker estimates provided by DLC and the avatar mesh coordinates (“automatic coding”). This allowed the metric calculation of AU activation intensity in terms of Euclidian distance from the AU-associated marker position at its neutral starting frame to its position at expression apex (Fig. 2C and detailed in Supplementary Table S1). The thus obtained automatic coding was compared to the initial manual coding by normalizing the scores for each automatically quantified AU between 0 and 1 and subsequently setting all values < 0.1 to 0 (no AU activation) and all other values to 1, thereby mapping the automatic coding onto the format of the manual coding. The agreement between automatic and manual coding could then be compared in the same way as the agreement between two human coders: $\:Agreement=\frac{2*Number\:of\:AUs\:agreed}{AUs\:automatic+AUs\:manual}$

The agreement between DLC-tracked real videos and manual coding was 0.72 and the agreement between avatar mesh coordinate trajectories and manual coding was 0.84. Using the minimum agreement of novice coders with certified coders of > 0.7, required to become a new certified MaqFACS coder, we considered the performance of the automatic coding acceptable. Therefore, the quantified automatic coding results were used for all subsequent analyses.

**Visual similarity**: Visual similarities among the different expressions of each real test identity and the naturalistic avatar with prototypical expressions were calculated as the pairwise Pearson correlations between the quantified FACS compositions of the videos (analogous to Micheletta et al. 2015).

### Data analysis

All data were analyzed using MATLAB R2024a and the MATLAB Statistics and Machine Learning Toolbox (MathWorks).

**Generalization tests**: The binomial cumulative distribution function was used to determine whether the relative choice frequency of the category-associated target for each test stimulus exceeded chance (0.25; one-sided *p* < 0.05). We grouped tests into families corresponding to distinct sub-questions: (1) generalization to novel real monkey videos, (2) prototypical avatar expressions, (3) degraded avatar variants, and (4) human expressions. Each family except family 4, which only contained data from one monkey, was additionally split into (a) tests for monkey 1 and (b) tests for monkey 2. Within family 2a and 2b, we applied a Bonferroni correction given the small number of comparisons. Within all other families, we applied a Benjamini-Hochberg false discovery rate (FDR) correction, given the larger number of tests and the more exploratory nature of these comparisons.

**Difference in expected choices according to visual similarity**: Expected choice frequencies were equated to the visual similarity matrix (correlations between FACS compositions) and the difference in expected choices was calculated by subtracting the visual similarity matrix from the observed relative choice frequency matrix. The overall Pearson correlation between the visual similarities and the choice frequencies for incorrect targets was calculated (diagonals of visual similarity autocorrelation and correctly chosen targets were excluded). 

**Multinomial Logistic Regression (MLR)**: To identify factors driving categorization, we fitted MLR models to the trial-level choices using the MATLAB *fitmnr* function. Predictors included: “Expression” (categorical) for unambiguous expressions or expression intensities of “Lipsmack”, “SBT” and “Threat” (i.e. morphing weights in the range of 0 to 0.75) for the ambiguous morphed expressions, stimulus monkey identity (“Identity”, categorical), stimulus monkey body weight (“Weight”, assumed to be associated with perceived strength), stimulus monkey averting the gaze away from the camera (“Avert”, scored manually: 0 = direct gaze, 1 = gaze aversion), and the quantified MaqFACS AU composition of each video were considered as predictors. To avoid multicollinearity among AUs, we omitted redundant AUs (AU8, AU9, AU25) and combined correlated AUs into the following composite predictors: “BrowRaise” (AU1_2), “BrowLow” (AU41), “TeethBare” (AU10 + AU12 + AU16), “LipPucker” (AU18i * (AD181 + 1)), “JawOpen” (AU26 + AU27), “EarsErect” (EAU1 + EAU2) and “EarsFlat” (EAU3). All numeric predictors were z-scored. Choice of the Neutral target was set as reference category and the log-odds of choosing each of the other categories relative to this reference were calculated. Models with different sets of predictors were compared based on the Deviance *D* (generalized residual sum of squares), which measures the goodness of fit compared to a saturated/perfect model (lower values indicating better fit): $\:D=-2\left(\mathrm{log}L(b1,y\right)-\:\mathrm{l}\mathrm{o}\mathrm{g}(bs,y))$, whereby *L*(*b*,*y*) represents the maximum value of the likelihood function for a model with the parameters *b. b1* contains the estimated parameters for model 1 and *bs* the estimated parameters for the saturated model. When model 1 has a subset of the predictors in model 2, $\:\varDelta\:D=D2-D1$ has a Chi^2^-distribution with degrees of freedom equal to the difference in the number of predictors in model 1 and model 2, allowing for the calculation of a *p*-value. If the *p* < 0.05, we considered model 2 significantly better than model 1 and included the additional predictor(s). Sample sizes pertain to the number of trials per subject.

**Random Forest Classification**: To clarify which (potentially non-linear) combinations of AU activation thresholds would lead to which categorization choice, we trained random forest classifiers (500 trees) on the trial-level target choice data from monkey 1 using the MATLAB *TreeBagger* function, considering the composite AU predictor set (see MLR) and the full set of AU predictors. Random forests are a robust, non-parametric ensemble learning method (Breiman 2001) increasingly used for classification problems in animal behavior research (Bergen et al. 2023), and have previously been applied to human facial AU data to classify facial expressions (Pu et al. 2015) or predict valence and arousal ratings (Zhang et al. 2024). Random forests aggregate predictions from multiple decision trees, each trained on a bootstrapped data sample with random feature subsets considered at each split. The out-of-bag (OOB) error is computed from predictions on data not included in each tree’s bootstrap sample, providing an internal validation metric. Feature importance was quantified using OOB permutation importance scores, which measure the increase in prediction error when each predictor’s values are randomly permuted. Decision rules were extracted through simplified decision trees *(fitctree)* and partial dependence plots, showing how category probabilities change as a function of individual predictor activation while holding the other predictors at their mean values.

**Pupil size**: Raw pupil size (vertical diameter) was preprocessed by first removing blinks (arbitrary eye tracker units < -4) and other artifacts larger or smaller than the mean +/- 2*standard deviation, then smoothing the signal with a 2nd order Savitzky-Golay filter of window size 100 ms and finally translating the signal within each block to *z*-scores. Average pupil size per trial from 1750 ms (when the pupillary light reflex to the movie onset was assumed to have settled) to 2750 ms was calculated, which was the unit for all statistical comparisons. The brightness of each test video frame was calculated as the mean pixel intensity of the grayscale image. The brightness between expressions was neither significantly different for the complete real expression set (Kruskal-Wallis ANOVA, *p* = 0.92) nor the incomplete real expression set missing the SBT (Kruskal-Wallis ANOVA, *p* = 0.69). All avatar videos had been rendered using the exact same lightning and scene settings for each expression. Pupil sizes between expression categories were compared using ANOVAs and *post-hoc* Tukey-Kramer tests. Post-hoc comparisons were based on estimated marginal means from the constrained (Type III) ANOVA model, which adjusts for unequal trial numbers across identities rather than weighting by raw cell sizes. General Linear Models (GLMs) were fitted to the pupil size data using the same stepwise approach and the same set of predictors as for the MLR models of the choice behavior, plus the additional predictor video brightness and the control predictors total AUs intensity (operationalized as the sum of all normalized (range 0, 1) AU scores per video) and number of activated AUs (operationalized as the number of AUs with normalized scores > = 0.1 per video, analogous to the operational definition of AU activation for the automatic to manual coding comparison). Sample sizes pertain to the number of trials per subject.

## Results

### Part I: Naturalistic monkey stimuli

#### Partial generalization of facial expression categorization to new videos with differential pupil reaction to the expression categories

**Fig. 3:**
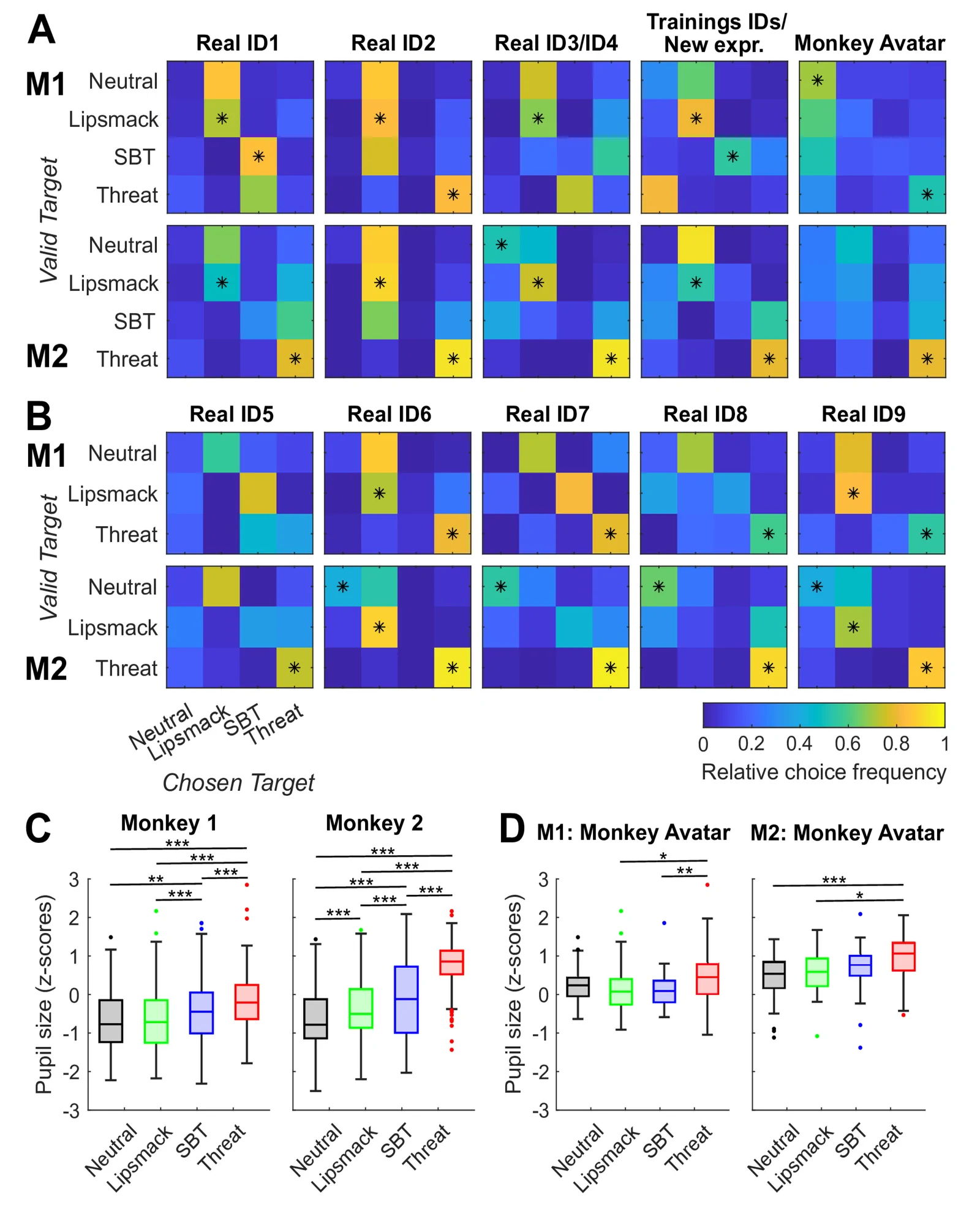
Expression categorization frequencies and pupil sizes for all pure test expressions (real monkeys and naturalistic monkey avatar) for monkey 1 (M1) and monkey 2 (M2), *N* = 50–60 trials per video per subject **(A)** Confusion matrices for new monkey identities (ID1-4), previously seen monkey identities with previously unseen expression categories (Training IDs/New expr.) and the naturalistic monkey avatar with prototypical expressions, complete expression repertoire. Asterisks indicate significant generalization (binomial tests, chance level 0.25, *p* < 0.05, FDR-corrected). **(B)** Confusion matrices for new monkey identities with incomplete expression repertoire, SBT missing (ID 5–9). **(C)** Boxplots of pupil sizes during the viewing of each expression category for all pure test expressions (real monkeys and naturalistic monkey avatar) with the complete expression repertoire *p < 0.05, **p < 0.01, ***p < 0.001 (ANOVA Expression x Identity, *post-hoc* Tukey-Kramer tests). **(D)** Boxplots of pupil sizes for the naturalistic monkey avatar separately. Systematic pupil size differences between real monkey videos and monkey avatar are influenced by differences in stimulus background brightness

After the two monkeys had been trained to categorize videos of conspecifics showing neutral, lip-smacking, silent bared-teeth (SBT) and open-mouth threat facial expressions, they could to some extent generalize the learnt categories to new monkey identities, including a naturalistic monkey avatar (Fig. 3A-B). The threat expression was largely correctly categorized and also the lip-smacking expression was often reliably categorized as such, however rarely distinguished from neutral expressions, whereas the classification of the SBT display varied the most. Overall, the correct classification of real expressions was moderate with 37.74% for monkey 1 (M1, neutral 10.72%, lip-smacking 53.46%, SBT 40.36%, threat 47.87%) and 50.76% for monkey 2 (M2, neutral 31.05%, lip-smacking 50.29%, SBT 12.98%, threat 87.71%). Similarly, the correct classification of the monkey avatar videos was 40.29% for M1 (neutral 70.18%, lip-smacking 22.03%, SBT 17.24%, threat 51.72%) and 36.99% for M2 (neutral 25.45%, lip-smacking 33.96%, SBT 8.93%, threat 79.63%) (Fig. 3A, right-most panel). A Multinomial Logistic Regression (MLR) analysis of the real monkey video and naturalistic avatar categorization choices with “Expression” as categorical predictor corroborated that the expression category significantly predicted the target choice (MLR Model 1 in Supplementary Fig. S4A and Supplementary Table S2).

Errors were not explained by FACS-based visual similarity between expressions alone, as the visual similarity, in terms of correlation between the FACS compositions of expression pairs, was not correlated with the choice frequency of an incorrect target, neither for monkey 1 (Pearson’s *rho* −0.13, *p* = 0.20), nor for monkey 2 (Pearson’s *rho* −0.053, *p* = 0.59). The differences between observed relative choice frequency of each target in response to each expression video and expected choice frequency according to the visual similarity between the shown expression and the expression associated with the selected target for each test identity are presented in Supplementary Fig. S2. If the monkeys selected a target as often as predicted by the visual similarity, the difference in expected choices would be zero (white), whereas the frequent positive (red) and negative (blue) values indicate that targets were selected more and less often as visual similarity would predict.

**Fig. 4:**
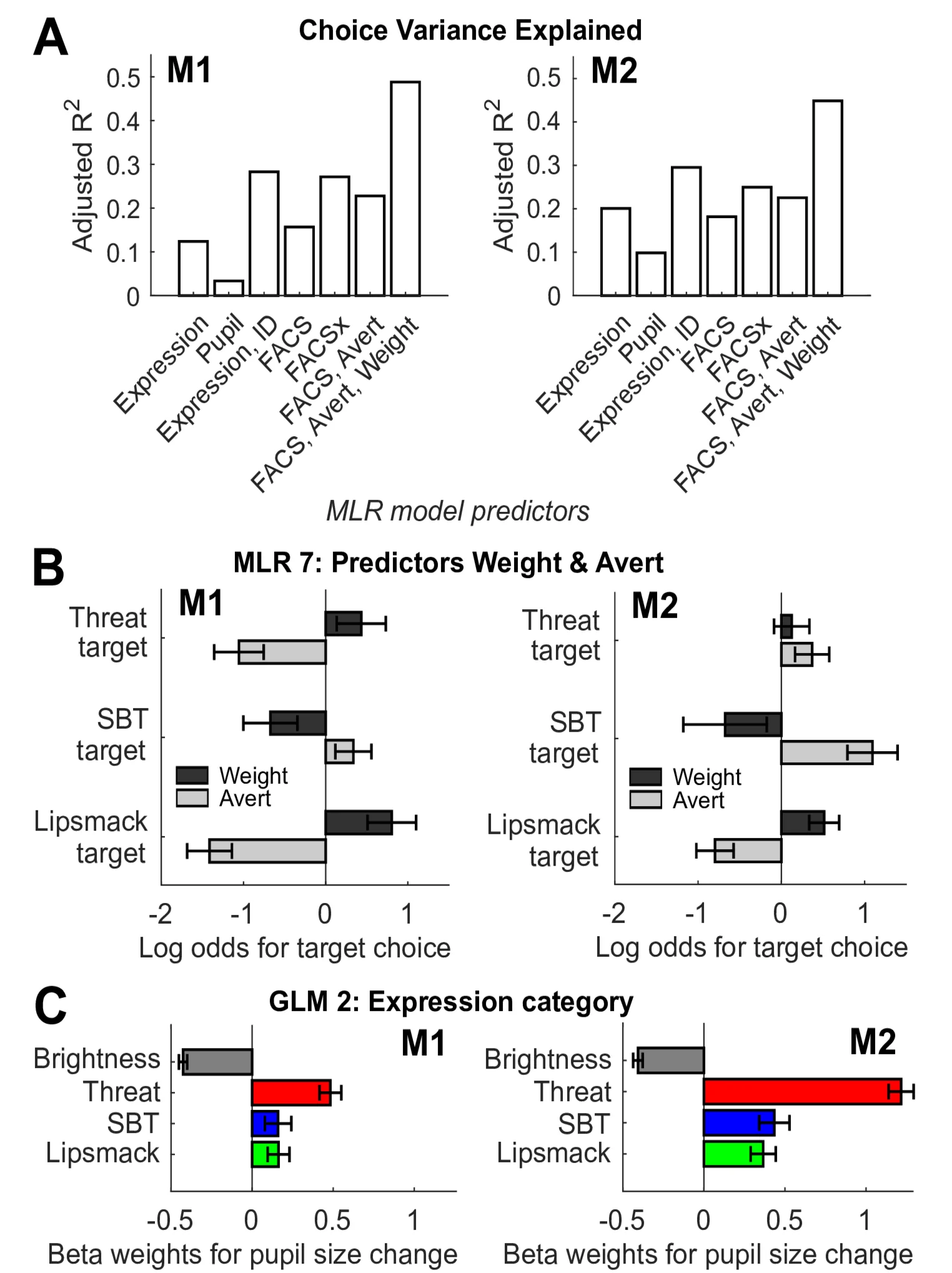
Models of choice and pupil size data in response to the pure (real and naturalistic monkey avatar) expression videos for monkey 1 (M1) and monkey 2 (M2). All model details are available in Supplementary Table S2 (choice) and Supplementary Table S3 (pupil size). Error bars represent 95% confidence intervals. **(A)** Proportion of the variance in choice behavior explained (adjusted R^2^) by MLR models with different sets of predictors. “FACS” stands for the composite FACS predictors BrowRaise (AU1_2), BrowLow (AU41), TeethBare (AU10 + AU12+AU16), LipPucker (AU18i*(AD181 + 1)), JawOpen (AU26 + AU27), EarsErect (EAU1 + EAU2) and EarsFlat (EAU3). “FACSx” stands for the composite FACS predictors + 3-way interactions between mouth predictors (LipPucker, TeethBare. JawOpen) x brow predictors (BrowRaise, BrowLow) x ear predictors (EarsErect, EarsFlat). **(B)** Log odds coefficients for choosing each target relative to choosing the Neutral target (reference category) for the predictors gaze aversion (Avert) and body weight (Weight) in MLR model 7; all model coefficients are plotted in Supplementary Fig. S4C. **(C)** Beta coefficients in GLM Model 2 with expression category and video brightness as predictors of the pupil size

Analysis of the pupil size during the stimulus presentation underlines the facilitated recognition of threat and suggests that the expression categories were perceived differentially despite partial lack of behavioral distinction, as pupil size was significantly different between the seen facial expression categories. Pupil size is a measure of autonomic arousal with larger pupils found in states of higher arousal (Grujic et al. 2024) and has been shown to change in reaction to socially important rhesus macaque stimuli (Ebitz et al. 2014). For all pure expressions with the full expression repertoire (real monkey videos and naturalistic monkey avatar), two-factorial ANOVAs demonstrated significant main effects of Expression and Identity, and a significant interaction of Expression x Identity (M1: Expression, *F*(3, 948) = 40.53, *p* < 0.001, Identity, *F*(3, 948) = 259.70, *p* < 0.001, Expression x Identity, *F*(9, 948) = 10.15, *p* < 0.001; M2: Expression, *F*(3, 953) = 318.20, *p* < 0.001, Identity, *F*(3, 953) = 256.97, *p* < 0.001, Expression x Identity, *F*(9, 953) = 45.83, *p* < 0.001). Pupils were overall largest, indicating highest arousal, when seeing threatening expressions, second largest when presented with the SBT or the lip-smacking display, and smallest when the stimulus monkey kept a neutral expression (Fig. 3C and Supplementary Fig. S3A; *post-hoc* Tukey-Kramer tests). Also for the naturalistic avatar alone, pupils were significantly enlarged when viewing the threatening expression (Fig. 3D). Similarly, two-factorial ANOVAs of the pupil sizes in response to the real monkey videos with incomplete expression repertoire confirmed this pattern and significant effects of Expression, Identity and their interaction (Supplementary Fig S3B; M1: Expression, *F*(2, 784) = 67.30, *p* < 0.001, Identity, *F*(4, 784) = 57.34, *p* < 0.001, Expression x Identity, *F*(8, 784) = 14.44, *p* < 0.001; M2: Expression, *F*(2, 845) = 272.04, *p* < 0.001, Identity, *F*(4, 845) = 107.01, *p* < 0.001, Expression x Identity, *F*(8, 845) = 22.52, *p* < 0.001). A General Linear Model (GLM) analysis of the pupillary reactions to the real monkey and naturalistic avatar expressions corroborated these results while explicitly accounting for the influence of video brightness (GLM Model 2 in Fig. 4C and Supplementary Table S3). Conversely, pupil size alone had a small but significant predictive effect for the choice behavior in an MLR: larger pupils predicted choice of the Threat target and smaller pupils predicted choice of the Lipsmack target. (MLR Model 2 in Supplementary Fig. S4B and Supplementary Table S2).

#### Interactions between face parts and additional social information influenced the categorization behavior

Examination of the categorization performances for each test identity of both subjects in Fig. 3A-B suggested that the monkeys used idiosyncratic categorization strategies for different test identities. This was confirmed by an MLR analysis with “Identity” added to “Expression” as predictors of the target choice, which significantly improved the model fit (MLR Model 3, M1: *ΔD* = 919.26, *p* < 0.001, M2: *ΔD* = 548.29, *p* < 0.001, Supplementary Table S2 and Fig. 4A). We therefore wondered what factors exactly in the faces and facial movements of the different test identities might have led to these differences in categorization. We hypothesized that subtle variations in the exact facial movement compositions (eyebrow and ear movements, degree of mouth movements), information about the monkey’s perceived strength, or direct vs. averted gaze might cause facial expressions to be perceived differently. To explore these hypotheses, we compared different MLR models in a step-wise procedure, the results of which are summarized in Supplementary Table S2 and Fig. 4A. In order to describe facial movements agnostic of a priori category labels, we used the MaqFACS framework to identify and quantify activated facial action units (AUs) and thus deconstruct the facial expressions into their muscle movement components. The composite FACS predictors (MLR Model 4, M1: *D* = 4443.02, M2: *D* = 4082.73) yielded similar explanatory power as the expression category predictor (MLR Model 1, M1: *D* = 4642.69, M2: *D* = 4009.70). However, including three-way interactions between the mouth predictors (“LipPucker”, “TeethBare”, “JawOpen”), the brow predictors (“BrowRaise”, “BrowLow”) and the ear predictors (“EarsErect”, “EarsFlat”), which we abbreviated as “FACSx”, yielded a significantly improved model (MLR Model 5, M1: *ΔD* = 682.59, *p* < 0.001, M2: *ΔD* = 415.35, *p* < 0.001). Moreover, the monkeys did not seem to judge only the shown expression, but rather interpret a facial display differently depending on whether the facial display included averted gaze and depending on the stimulus monkeys’ weight (assumed to be associated with perceived strength), as adding the predictors “Avert” (MLR Model 6, M1: *ΔD* = 384.43, *p* < 0.001, M2: *ΔD* = 226.61, *p* < 0.001) and “Weight” (MLR Model 7, M1: *ΔD* = 1391.58, *p* < 0.001, M1: *ΔD* = 1132.10, *p* < 0.001) both yielded significantly improved models, evaluated by decreased Deviance *D*. Higher body weight significantly decreased the probability that the SBT target would be chosen. Averting of the gaze significantly decreased the probability for the choice of the Lipsmack target and increased the probability for the choice of the SBT target, whereas the direction of the effect for the Threat target was inconsistent (Fig. 4D and Supplementary Fig. S4C). We wished to add those predictors to MLR Model 5 in order to obtain a model of maximal explanatory power, however, this led to problems with model convergence. Therefore, the predictors Avert and Weight were added to MLR Model 4.

Identical stepwise GLM analysis was performed for the pupil size whilst taking video brightness into account for all models (Supplementary Table S3). Analogously to the choice behavior, the pupil size was similarly well explained by expression category (GLM Model 2, M1: *D* = 668.82, M2: *D* = 930.32) and FACS composition (GLM Model 4, M1: *D* = 661.30, M2: *D* = 968.72), and model fit was improved by accounting for interactions between the mouth, brow and ear AU predictors (GLM Model 5, M1: *ΔD* = 50.82, *p* < 0.001, M2: *ΔD* = 142.32, *p* < 0.001), as well as by adding the predictors “Avert” (GLM Model 6, M1: *ΔD* = 34.46, *p* < 0.001, M2: *ΔD* = 94.86, *p* < 0.001) and “Weight” (GLM Model 7, M1: *ΔD* = 145.49, *p* < 0.001, M2: *ΔD* = 246.05, *p* < 0.001) to MLR Model 4. Control analyses using the total intensity of AUs in the video (GLM Model 8, M1: *D* = 679.96, M2: *D* = 1141.83) or the number of activated AUs (GLM Model 9, M1: *D* = 710.63, M2: *D* = 1185.33) as predictors yielded higher Deviance *D* values than models employing the expression category or the FACS composition as predictors, indicating worse model fit and showing that the amount of motion per se did not drive arousal.

### Part II: Synthetic stimuli (avatar variants)

#### Categorization of morphed expressions reflected their position in morphing space and pupil size reflected threat intensity

**Fig. 5:**
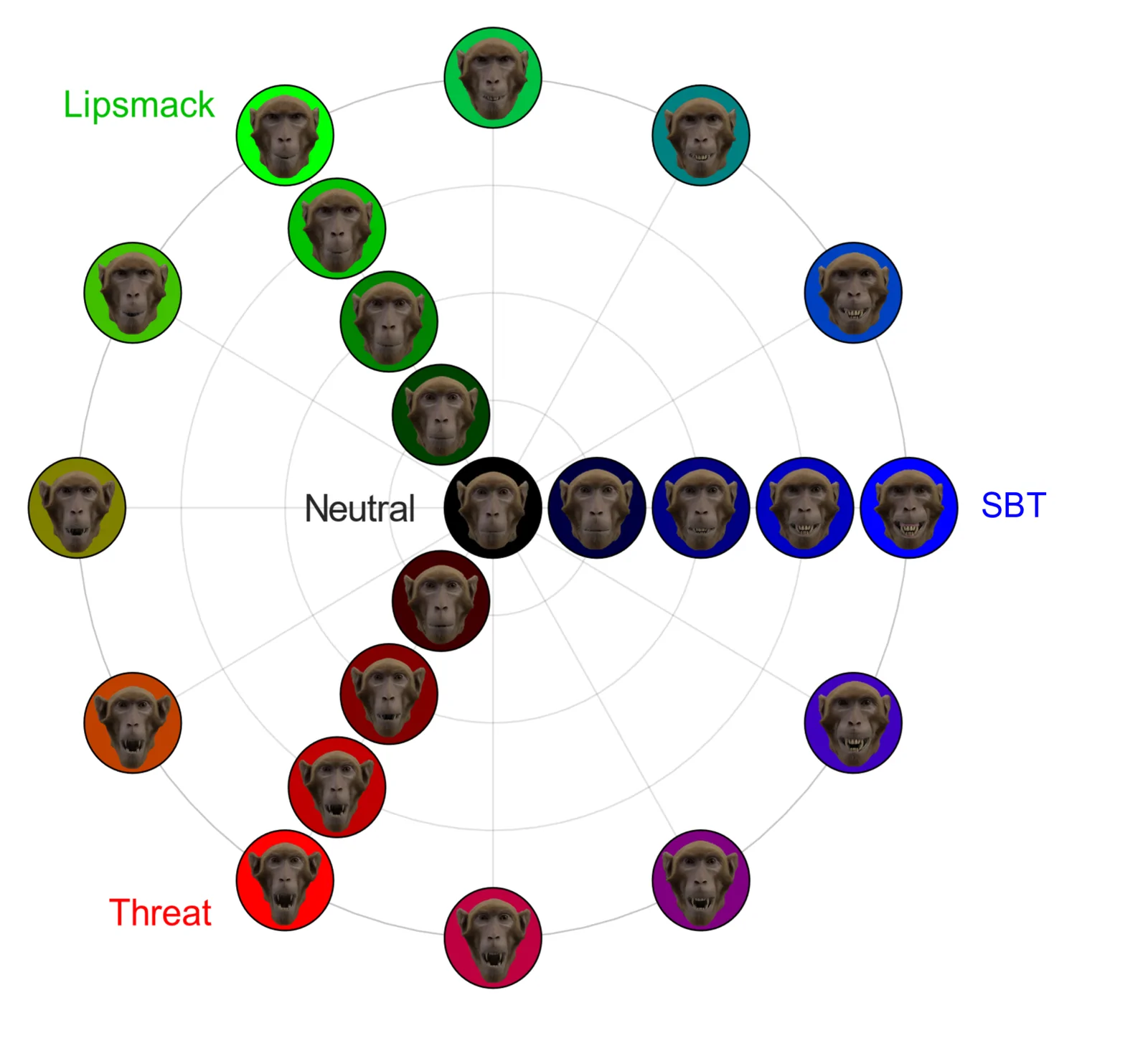
Monkey avatar expression space, comprising the pure, motion capture-based expressions neutral, lip-smacking, silent bared-teeth display (SBT) and open-mouth threat, and graded morphs in steps of 25% between two pure expressions

**Fig. 6:**
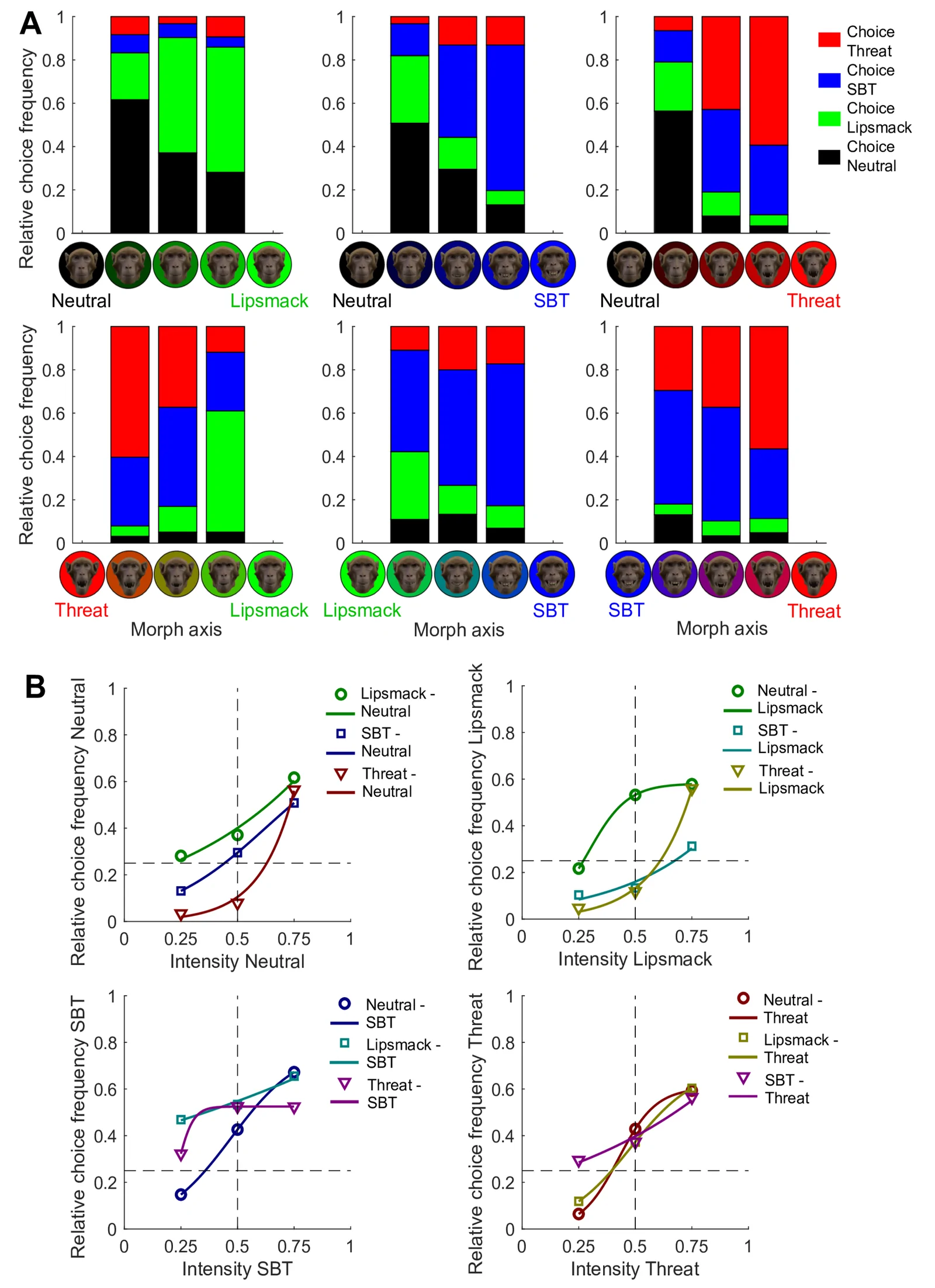
**(A)** Relative choice frequencies of monkey 1 for all morphed avatar videos, shown for each morph axis between the prototypical expressions Neutral, Lipsmack, SBT and Threat, *N* per video = 53 trials. **(B)** Relative choice frequencies (monkey 1) of the Neutral-, Lipsmack-, SBT- and Threat-associated targets for each morph axis, plotted against the intensity of the associated expression in the morphed video. Horizontal dashed lines indicate the chance choice levels of 0.25, vertical dashed lines indicate the 0.5 expression intensity levels. Logistic fits are overlaid

Having found that facial expression perception was influenced by simultaneous eyebrow and ear movements, but also by factors beyond the expression itself, namely depending on who is showing the expression and where exactly the individual is looking, we investigated with a fully controlled avatar stimulus set, (1) which combination of facial movements lead to the perception of a facial expression category, and (2) which facial shape and appearance features are necessary and sufficient for a facial movement to be perceived as falling into one of the trained facial expression categories. Before this second test phase, the pure, prototypical facial expression videos of the naturalistic avatar were added to the training stimulus set and the monkeys were briefly trained again until they could reliably categorize the avatar videos. To answer question 1), we generated a stimulus set of morphed expressions (depicting one identity with constant direct gaze), whereby expressions were interpolated continuously between two prototypical facial expressions in steps of 25% intensity (Fig. 5). The target choice frequencies, depicted in Fig. 6 (monkey 1) and Supplementary Fig. S5A (monkey 2) show that the categorization of the morphed avatar videos followed the morph axes, i.e. the stronger a facial expression category was represented in a video, the more frequently the video was classified as belonging to this category. Clearer lip-smacking features, compared to SBT or Threat features, seemed necessary though for an expression to be categorized as Lipsmack, particularly along the Lipsmack – SBT morph axis, and a general preference for the SBT target was apparent.

**Fig. 7:**
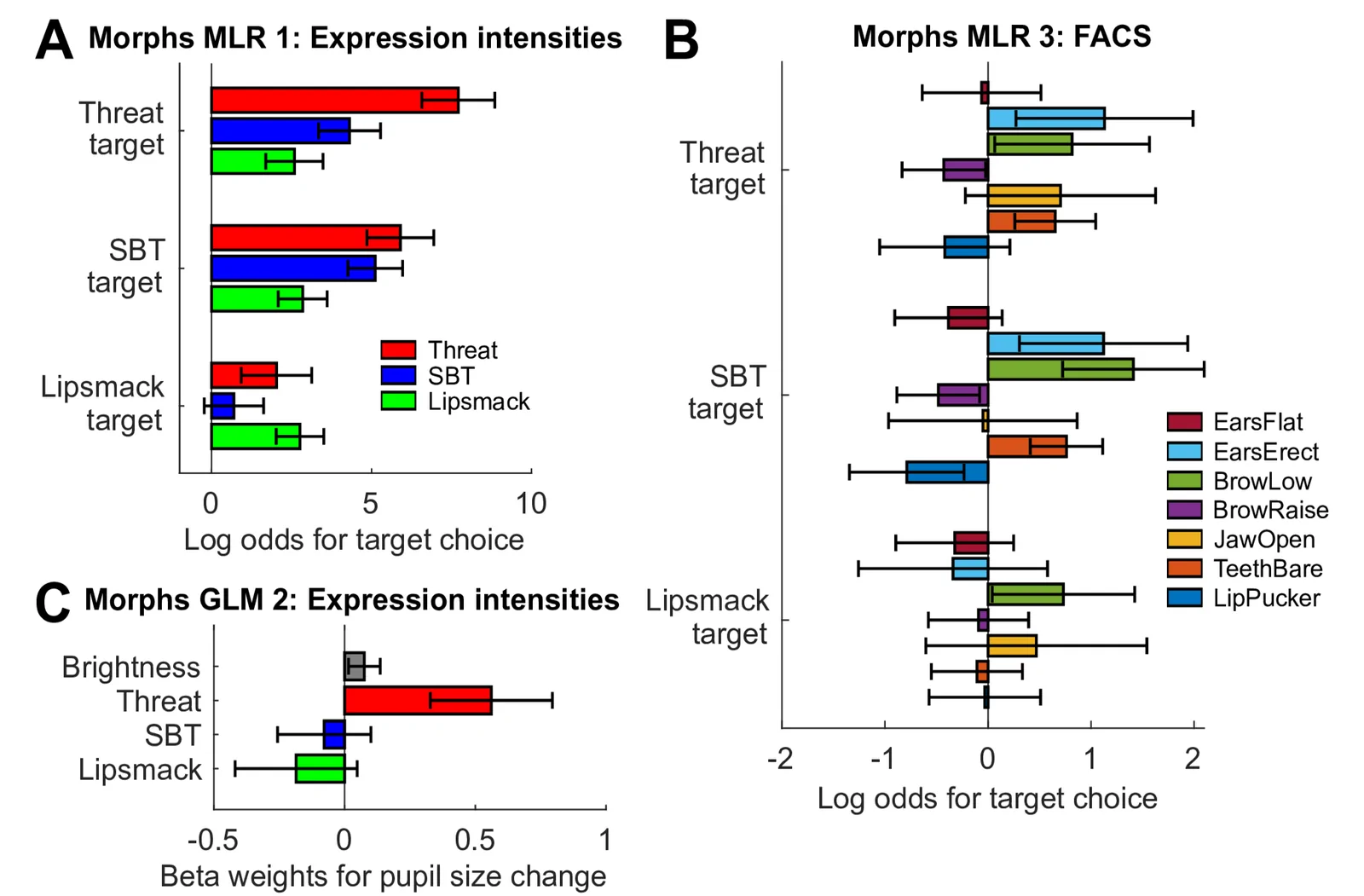
Select models of choice and pupil size data in response to the morphed avatar expressions for monkey 1. All model details are available in Supplementary Table S4 (choice) and Supplementary Table S5 (pupil size). Error bars represent 95% confidence intervals. **(A)** Log odds coefficients for choosing each target relative to choosing the Neutral target (reference category) in Morphs MLR Model 1 with expression intensities in the morphed expressions as predictors. **(B)** Log odds coefficients in Morphs MLR Model 3 with the composite FACS predictors. **(C)** Beta coefficients in Morphs GLM Model 2 with expression component intensities in the morphed expressions and video brightness as predictors of the pupil size

Given the limited data coverage for monkey 2 for the morphed avatar expressions, the following results focus on data from monkey 1; results for monkey 2 are provided as Supplementary Figures while Supplementary Table S4 summarizes MLR analyses for both subjects. The first MLR analysis showed that monkey 1’s choice behavior was very similarly well predicted by the intensities of “Lipsmack”, “SBT” and “Threat” in the morphed expression (Morphs MLR Model 1: *D* = 2431.90, Fig. 7A) as by the FACS composition (Morphs MLR Model 3: *D* = 2409.21, Fig. 7B), which suggests that the category transitions were well captured by the AU metrics. Adding two-way interaction terms to the expression intensities yielded only a small improvement (Morphs MLR Model 2: *ΔD* = 27.80, *p* < 0.001). When attempting to add interaction terms to the FACS AU predictors, analogously to the analysis in part I, maximum likelihood estimation failed to converge because the design matrix was nearly singular, indicating multicollinearity in the predictor matrix, likely resulting from lack of feature variance among the standardized morphed expressions. The same pattern of results was observed for monkey 2 (Supplementary Fig. S6).

Analogous GLM analysis of the pupil size under inclusion of video brightness as predictor yielded small, but significant effect sizes for monkey 1 and monkey 2 (Supplementary Table S5). Again, expression component intensities (Morphs GLM Model 2: *D* = 424.19) explained monkey 1’s pupil reactions similarly well as the FACS composition of the video (Morphs GLM Model 4: *D* = 421.50), but considering interactions among the expression intensities did not significantly improve the model (Morphs GLM Model 3: *ΔD* = 1.14, *p* = 0.77), and trying to add interaction terms to the FACS model again resulted in a rank deficient predictor matrix. As evident from Morphs GLM Model 2 in Fig. 7C (monkey 1) and Supplementary Fig. S6C (monkey 2), and from pupil size swarm plots in Supplementary Fig. S5B, pupils were larger the higher the intensity of threat components in an expression, while Lipsmack and SBT component intensity in the morphed expressions did not consistently influence pupil size to one direction. However, trends were consistent across the two subjects. Control analyses using the total intensity of AUs in the video (Morphs GLM Model 5: *D* = 461.04) or the number of activated AUs (Morphs GLM Model 6: *D* = 460.40) as predictors did not explain the pupil size better than video brightness alone, and markedly worse than the expression intensities or the FACS composition, which shows also for the morphed expressions that motion intensity per se did not increase arousal.

#### Eyebrow and ear movements influenced the categorization of ambiguous expressions

**Fig. 8:**
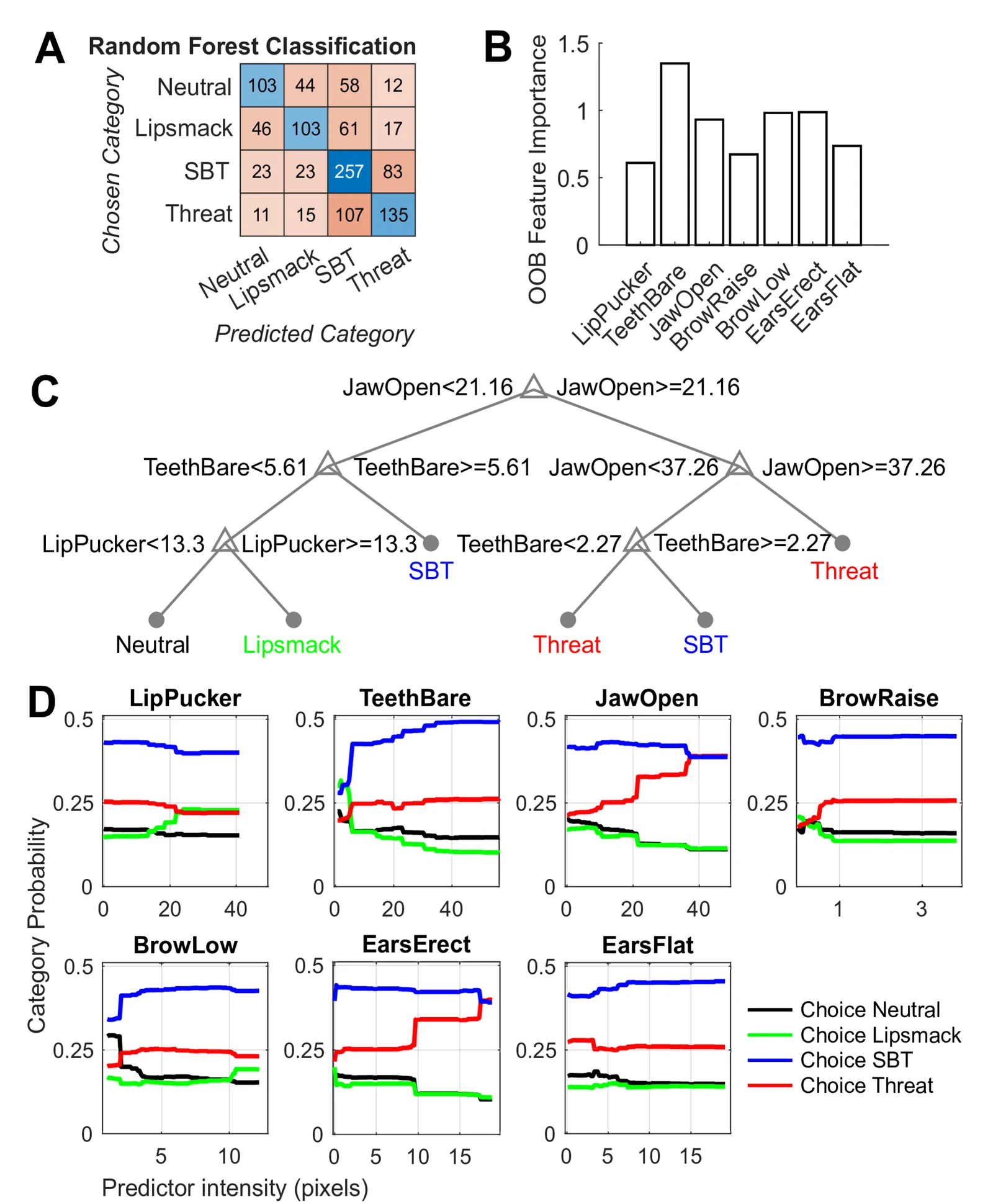
Random Forest Classification results of the morphed avatar expression categorizations by monkey 1 using the composite FACS predictors “BrowRaise”, “BrowLow”, “TeethBare”, “LipPucker”, “JawOpen”, “EarsErect” and “EarsFlat” (composite model). **(A)** Heatmap showing the predicted category vs. the chosen category. **(B)** Out-of-bag feature importance of the predictors. **(C)** Simplified decision tree elucidating which combinations of predictor activation intensities lead to which category choices. **(D)** Partial dependency plots showing how individual predictor intensities influence the probability for a category to be chosen

To further elucidate which facial movement features decided categorization choices at which threshold, we trained Random Forest Classifiers (RFC), which are based on decision trees and thus naturally handle multicollinearity, complex interactions and non-linear relationships among predictors without the need for explicit modeling. All RFC analyses were conducted on the choice data from monkey 1 only, as insufficient data was available from monkey 2. The first RFC used the same set of composite FACS predictors as for the MLR analysis (composite model) to predict the categorization of the morphed expressions. Classification accuracy of 54.5% (OOB error 0.455) exceeded chance level (25%) and was 19.3% better than a baseline model only predicting the most frequently chosen category (the SBT target). This modest prediction accuracy is expected given the ambiguous nature of morphed expressions near category boundaries. Classification results are shown in Fig. 8A.

Given the robustness of tree-based methods against multicollinearity – correlated predictors simply compete for inclusion rather than inflating each other’s apparent importance, leaving predictions unbiased (Breiman 2001) – we asked whether the full set of AUs might yield better predictions and compared the composite model to a model using the full set of individual AU predictors (full AU model). We obtained equal prediction accuracy with the full AU model (OOB error 0.455, Supplementary Fig. S8A), therefore we focus on the results from the more parsimonious composite model, which also facilitated comparison with the MLR findings.

Our analysis revealed TeethBare as the most important feature (Fig. 8B), which was used in 17.1% of splits. The most frequent root split was EarsErect in 26.0% of trees, closely followed by TeethBare (25.2%) and JawOpen (22.2%). Similarly, the most important feature in the full AU model was AU25 (lips parted) (Supplementary Fig. S8B), used in 10.9% of splits and as most frequent root split in 29.6% of trees, which makes sense as parted lips are involved both in teeth baring and jaw opening, but limits the diagnostic value of AU25 for distinguishing between SBT and threat expressions, which is why we omitted it from the set of composite predictors. A simplified decision tree for the composite model (accuracy 54.5%) shows that given large, but ambiguous degrees of jaw opening, any noticeable teeth baring ( > = 2.27 pixels) led to the selection of the SBT target over the Threat target, and given small degrees of both jaw opening and teeth baring, lip puckering ( > = 13.33 pixels) decided the choice of the Lipsmack target instead of the Neutral target in our morphed stimulus set (Fig. 8C). A simplified decision tree for the full AU model detailed that raised eyebrows helped distinguish Lipsmack from Neutral and SBT and lowered eyebrows contributed to the distinction of SBT from Threat (Supplementary Fig. S8C). A partial dependency plot for the composite model prompts similar conclusions. Partial dependency plots elucidate how category choice probabilities change as a function of a single predictor value. Sharp category probability transitions occurred expectedly in TeethBare (21.3% increase for the SBT target and 21.8% decrease for the Lipsmack target), JawOpen (17.6% increase for the Threat target) and LipPucker (8.7% increase for the Lipsmack target), but also in EarsErect (18.2% increase for the Threat target), BrowRaise (8.2% increase for the Threat target) and BrowLow (9.6% increase for the SBT target and 14.1% decrease for the Neutral target) (Fig. 8D). These findings confirm MLR results indicating that brow and ear predictors also significantly influence category choices in addition to mouth predictors (Morphs MLR Model 3, Fig. 7B).

In order to compare the observed feature importance in the morphed expressions to the unambiguous expressions, we also fitted the composite model to the categorization choices of monkey 1 for all pure expression videos (real monkey videos and naturalistic avatar). Trained with the categorizations of these unambiguous expressions, model accuracy increased to 69.8% (OOB-error 0.302, Supplementary Fig. S9A), 29.3% better than a baseline model predicting only the most common category in this data set (the Lipsmack target). As for the morphed expressions, TeethBare was the most important feature (Supplementary Fig. S9B), used in 20.2% of all splits and 38.0% of root splits. Comparable probability changes depending on individual predictor values (Supplementary Fig. S9D) and examination of a simplified decision tree (Supplementary Fig. S9C) again suggested that raised brows led to the choice of Threat over SBT, and lowered brows helped the distinction between Neutral and Lipsmack.

A control analysis using an RFC model with the relative intensities of Lipsmack, SBT and Threat in the morphed expressions as predictors yielded identical prediction accuracy as the composite and full AU models (OOB error 0.455, Supplementary Fig. S10A) and identified Threat intensity as the most important feature (Supplementary Fig. S10B) and most frequent root split (69.0% of trees). A simplified decision tree for this model (Supplementary Fig. S10C) confirms that the degree of jaw opening can be equated to Threat intensity, the degree of teeth baring to SBT intensity, and the degree of puckered lips to Lipsmack intensity. Partial dependency plots (Supplementary Fig. S10D) corroborate what choice frequencies (Fig. 6) indicated: the largest category probability increases occurred between 0.25 and 0.5 expression intensity, with additional smaller increases between 0.5 and 0.75.

#### Facial expression recognition required biologically plausible texture, but not fully realistic monkey faces or coherent motion

In order to further dissect which attributes are necessary for reliable facial expression perception, we first tested versions of the prototypical avatar expressions with time-scrambled frame order, thus abolishing temporal continuity of the movement. Next, we manipulated the facial shape and appearance in two ways: On the one hand, we reduced the realism of the avatar in a stepwise manner while keeping the same movement pattern as for the prototypical expressions of the naturalistic avatar. We first removed the fur (furless avatar), then the color (gray avatar), then the texture (wire avatar), and finally all discernable facial shape (form-from-motion point-light avatar). On the other hand, we changed the facial shape and appearance completely by changing the species of the avatar head from monkey to human, while displaying the exact same monkey facial expressions.

A two-way ANOVA of monkey 1’s pupil size with factors Expression and AvatarType revealed significant main effects of Expression, *F*(2, 1397) = 5.31, *p* < 0.001, and AvatarType, *F*(4, 1397) = 1564.72, *p* < 0.001, and a significant interaction of Expression x AvatarType, *F*(14, 1397) = 3.49, *p* < 0.001. Given this interaction effect and the fact that the effect of AvatarType cannot be disentangled from systematic differences in brightness between the different avatars, we conducted post-hoc pairwise comparisons (Tukey-Kramer tests) to investigate expression differences within each avatar type separately.

**Fig. 9:**
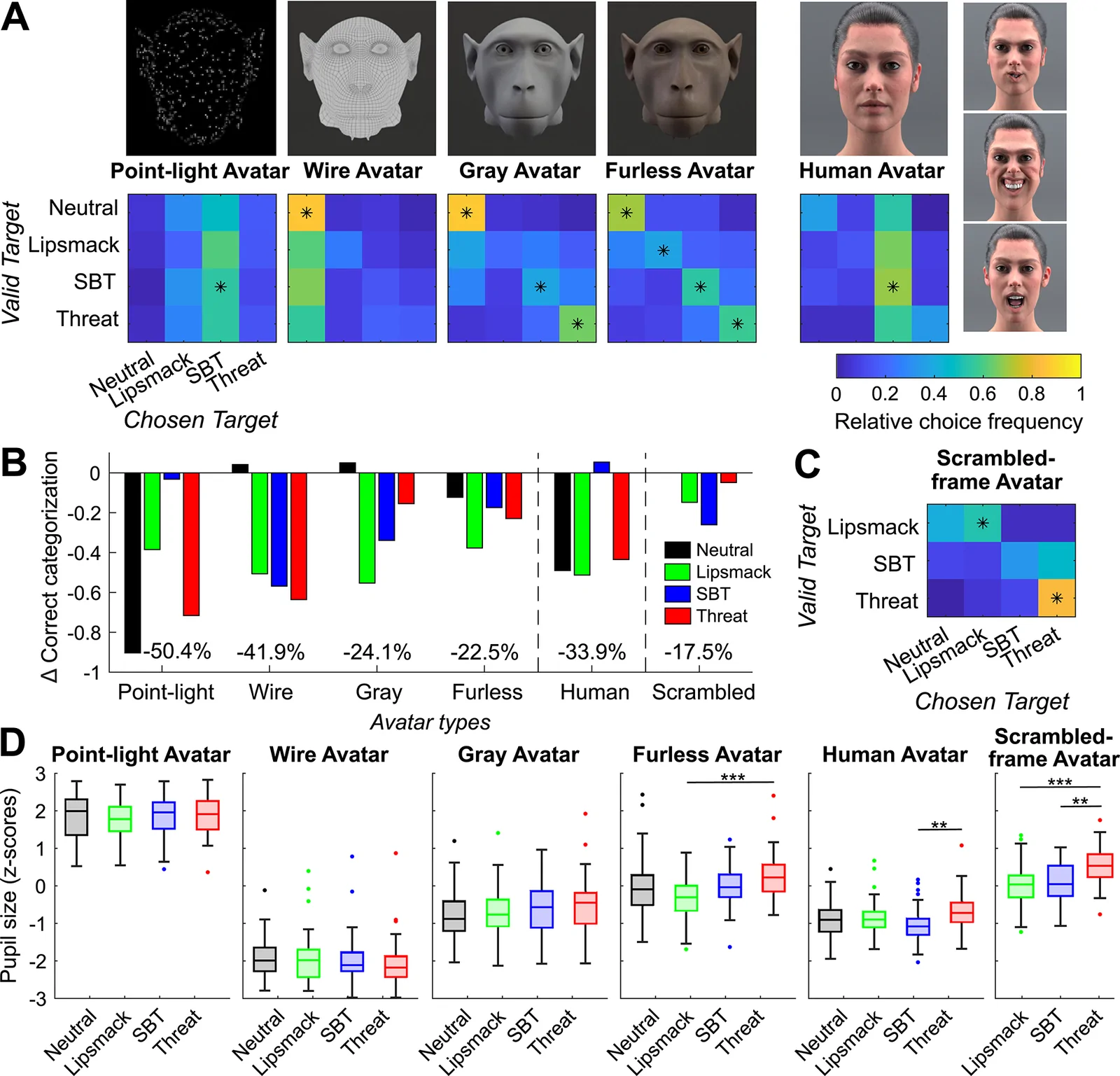
**(A)** Expression categorization frequencies of different avatar variants for monkey 1, asterisks indicate correct categorization significantly above chance level (binomial tests, *p* < 0.05, FDR-corrected). The first four avatar types increase in realism: point-light expressions (*N* per video = 60 trials), wireframe mesh (*N* per video = 53 trials), grayscale avatar (*N* per video = 53 trials) and furless avatar (*N* per video = 53 trials). Additionally, a human avatar with monkey expressions (*N* per video = 59 trials) was tested; peak lip-smack, SBT and threat expressions (top to bottom) are depicted next to the confusion matrix. **(B)** Difference in fraction of correct categorizations of avatar variants relative to the (now trained) naturalistic avatar per expression category for monkey 1. Percentages at the bottom indicate the overall difference in correct categorizations for each avatar type. **(C)** Expression categorization frequencies for the naturalistic monkey avatar with time-scrambled frame order for monkey 1 (*N* per video = 60 trials, binomial tests, *p* < 0.05, FDR-corrected). **(D)** Boxplots of pupil sizes of monkey 1 during the viewing of each expression category for all avatar variants. *p < 0.05, **p < 0.01, ***p < 0.001, (ANOVA, *post-hoc* Tukey-Kramer tests). Systematic pupil size differences between avatar variants are influenced by differences in avatar type brightness

The scrambled-frame avatars were still well recognized (Fig. 9C and B, right-most panel), while eliciting pupil reactions comparable to the prototypical monkey avatar and real monkey videos, with arousal increasing from Lipsmack over SBT to Threat (Fig. 9D, right-most panel). While the furless and gray avatars were reasonably well categorized, the wire avatar without texture, consisting only of the wireframe mesh surface, and the point-light avatar were not categorized, instead the monkey tended to select only one target for these avatar types (Fig. 9A and B). This behavioral pattern is corroborated by the pupil size, which exhibited significant expression-dependent modulation for the furless avatar, but not for the wireframe avatar or the point-light avatar (Fig. 9D). The limited data from monkey 2 suggests a similar pattern of categorization and pupil responses (Supplementary Fig. S10). We note that monkey 2 seemed able to discern the Threat expression of the wireframe avatar, possibly due to the conspicuous downward motion of the jaw for this expression, in the absence of a differential pupil reaction. While correct categorization of monkey expressions displayed by a human avatar was limited (Fig. 9A and B), significant pupil size modulation between expressions was observed (Fig. 9D).

#### Human facial expressions did not fit into the trained categories

**Fig. 10:**
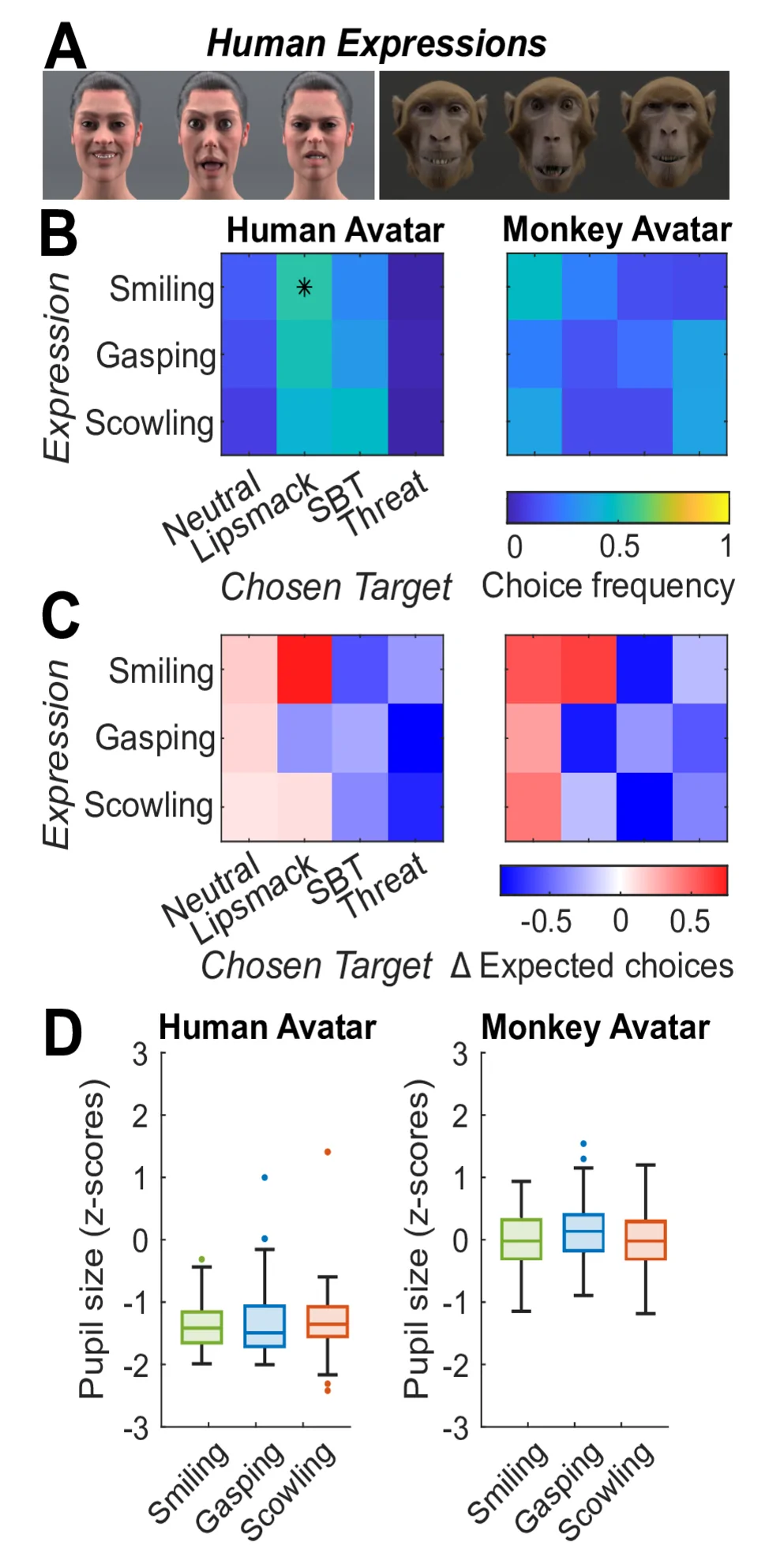
**(A)** Smiling (happy), gasping (fearful) and scowling (angry) human expressions of the human avatar (left) and the monkey avatar (right). **(B)** Expression categorization frequencies of monkey 1 for the human avatar and the monkey avatar displaying the human facial expressions smiling, gasping and scowling (*N* per video = 60 trials). The Lipsmack, SBT and Threat targets, respectively, were considered the “valid” according to putative homologous meanings. Asterisks indicate generalization significantly above chance level (binomial tests, *p* < 0.05, FDR-corrected). **(C)** Difference matrices calculated between observed relative choice frequency and the expected choice frequency according to the visual similarity between the human test expression and the monkey expression associated with the selected target. A value of zero (white) indicates that the target was selected as often as predicted by the visual similarity, positive values (red) indicate that the target was selected more often, and negative values (blue) indicate that the target was selected less often. **(D)** Boxplots of pupil sizes of monkey 1 during the viewing of each human expression category for the human and monkey avatars. Systematic pupil size differences between human avatar and monkey avatar are influenced by differences in stimulus and background brightness, compare (A)

We also tested how human facial expressions, displayed by the human avatar or the monkey avatar (Fig. 10A), would be categorized, in order to explore the possibility of a homologous interpretation across primate facial expressions. These stimuli could only be tested with monkey 1. We did not find a systematic categorization pattern of the human facial expressions. They were neither categorized according to putative homologous meanings (smiling/happy and lip-smacking, gasping/fearful and SBT, scowling/angry and threat), nor according to potential phylogenetic homology, such as SBT displays and human smiles (Fig. 10B). Moreover, the target choice frequencies were uncorrelated with the visual similarities between the human expressions and the target-associated monkey expressions, measured as the correlation between the FACS compositions of each expression pair (Supplementary Fig. S2A), for the human avatar (Pearson’s *rho* 0.05, *p* = 0.89) and the monkey avatar (Pearson’s *rho* −0.34, *p* = 0.29). The differences between observed relative choice frequency of each target and expected choice frequency according to the visual similarity between the viewed human expression and the target-associated monkey expression are depicted in Fig. 10C. These findings suggest that the human facial expression did not carry meaning for the subject monkey, a notion that is supported by the lack of differential pupil reactions between expressions (Fig. 10D). A two-factorial ANOVA of the pupil size showed neither a main effect of Expression (*F*(2, 381) = 1.53, *p* = 0.22), nor an interaction between Expression and AvatarType (*F*(2, 381) = 1.36, *p* = 0.26), only a significant main effect of AvatarType (*F*(1, 381) = 778.18, *p* < 0.001), likely due to differences in stimulus and background brightness between the human and monkey avatars.

## Discussion

We investigated how two rhesus macaques categorized facial expressions, and which facial features enabled expression perception. In a forced-choice categorization paradigm with neutral, lip-smacking, silent bared-teeth (SBT) and open-mouth threat displays, they successfully applied learned categories to unfamiliar individuals and avatars. Pupil size measurements showed graded physiological responses across expression types, demonstrating that the macaques extracted behavioral meaning rather than simply sorting low-level visual patterns. When viewing expressions morphed between two prototypes, categorization frequencies tracked the proportional intensity of each expression component, indicating perception along a continuum. To quantify similarity between expressions, we developed a novel automated approach combining DeepLabCut landmark tracking with the MaqFACS framework, usable without training as a MaqFACS-coder, enabling objective, muscle-based quantification of all facial movements in frontal videos of monkey faces without being limited to mouth movements only (Blumrosen et al. 2017) or requiring pre-labeled training datasets (Morozov et al. 2021). This approach showed that FACS-based visual similarity between expressions alone did not explain error patterns. Instead, categorization was influenced by how facial regions (mouth, eyebrows, ears) moved in coordination, whether gaze was averted, and by signaler characteristics exemplified by the displaying individual’s body weight. This suggests that the tested expression types were not interpreted as rigid categories with one meaning, but instead category-defining movements were interpreted in a nuanced manner embedded within their entire facial and signaler contexts. Systematic manipulation of avatar properties showed that neither temporally coherent motion, nor photorealistic rendering or a conspecific face were essential for expression recognition, but discernible facial shape and surface texture were required. Human expressions displayed by human and monkey avatars failed to elicit systematic categorization or differential arousal in the one tested macaque.

### Categorization of facial expressions did not follow clear morphological categories

In part I, we show that two rhesus macaques trained to categorize facial expressions into neutral, lip-smacking, silent bared-teeth (SBT) and open-mouth threat displays could to some extent generalize these learned categories to new instances of those expressions presented by new agents, both real monkeys and monkey avatars. Critically, viewing these expressions was accompanied by differential arousal as measured by pupil size, with incremental pupillary responses to neutral, lip-smacking, SBT and threatening faces. Addressing our first hypothesis regarding discrimination strategy, the differential physiological arousal precludes that the macaques resorted to the categorization of elementary facial movement features without attaching any meaning to the expressions. If they had relied solely on low-level visual features, we would have expected comparable performance across expression categories without physiological differentiation. Instead, the pattern of arousal responses, which was not explained by motion intensity per se, particularly the robust arousal to threatening faces, suggests that these videos were perceived as meaningful categories rather than arbitrary visual patterns. However, the categorization patterns did not align with a perception of the expressions as clear, mouth-movement-defined morphological categories, as miscategorization frequencies differed vastly between expressions, but were not correlated with the visual similarity between expressions as measured by the correlation between their FACS compositions. Open-mouth threat was most frequently recognized as such, reliably differentiated from lip-smacking and elicited the strongest arousal, confirming previous reports of heightened arousal when seeing threatening faces (Dal Monte et al. 2015; Landman et al. 2014; Siebert et al. 2020) and conceivably resulting from an evolutionarily conserved predisposition for prioritized processing of threatening faces (e.g. Frischen et al. 2008; Gong and Smart 2021; Kawai et al. 2016; Öhman et al. 2001; Sackett 1966). However, note that human data have suggested contesting views (e.g. Becker et al. 2011; Nummenmaa and Calvo 2015; Savage et al. 2013). Lip-smacking, a behavior usually observed in affiliative or appeasing contexts, was correctly categorized for most test monkey identities, but neutral expressions were frequently miscategorized as lip-smacking despite lacking any lip-smacking movement elements, confirming categorization based on factors beyond simple motion patterns. Finally, the categorization of the SBT display varied substantially across test identities. Using multinomial logistic regression (MLR), we explored which factors might explain these identity-dependent categorization patterns and identified the stimulus monkey body weight, gaze direction (direct vs. averted) and differential interactions between mouth, eyebrow and ear movements as significant predictors within our stimulus set.

After integrating mouth, eyebrow and ear movements, gaze direction and signaler characteristics such as body weight, which criterion could the macaques have relied on when categorizing the stimuli? One possibility is that they evaluated the facial displays based on the expressed putative internal states. Ever since Darwin (1872) systematically described facial expressions and proposed that they might relate to inner states, facial behavior in humans has been directly linked to the expression of discrete emotions and widely assumed to be universal (Ekman 1973, 1992; Ekman and Friesen 1971; Izard 1994; Russell 1994). This notion has only recently been questioned, with growing acknowledgement of the amenability of facial expressions to cultural learning and modulation by individual variation and context (e.g. Barrett et al. 2019; Durán and Fernández-Dols 2021; Le Mau et al. 2021; Valente et al. 2018). Also in macaques, facial expression processing skills have been shown to be positively influenced by early maternal care (O’Callaghan et al. 2025). Considering that macaques as nonverbal animals lack semantic emotion concepts, they might sort facial expression images similarly to human dementia patients, who have lost semantic emotion concepts, categorizing them by valence into negative, positive and neutral faces (Lindquist et al. 2014). If the macaques classified the videos according to expressed affect, this would predict a clear separation of positive (presumably lip-smacking) and neutral faces, contrary to our observations. Another possibility is that they sorted the videos according to perceived behavioral intent (Crivelli and Fridlund 2019; Fridlund 1994; Frijda and Tcherkassof 1997; Waller et al. 2017), similarly to a crested macaque (*M. nigra*) that was able to predict social interaction outcomes from facial expressions (Waller et al. 2016). The Relational Model of conflict resolution distinguishes tolerant, avoidant and aggressive intent in social decision making (de Waal and Aureli 1997). This would explain the lack of differentiation between neutral faces and lip-smacking, as both might convey tolerance of the other, whereas the open-mouth threat display appears to unequivocally convey aggression. The SBT display with exposed teeth and pulled back lip corners has traditionally been described as negatively valued in the majority of anthropoid species, including the rhesus macaque, associated with fleeing, submission or subordination, which can be summarized as avoidant intent (de Waal and Luttrell 1985; Maestripieri 1997; Preuschoft 2000; van Hooff 1967), and which has brought forth the alternative name “fear-grin”. However, the SBT display is used in more positive contexts in some species, like Tonkean macaques (*M. tonkeana*) (Thierry et al. 1989), bonobos (Vlaeyen et al. 2022) and humans (the “smile”), where it signals affiliation, appeasement or pleasure, but also reward or dominance, with considerable cultural variation in humans (Martin et al. 2017; Rychlowska et al. 2015). Preuschoft and van Hooff (1995) proposed a power asymmetry in the use of this expression across macaque species: While despotic species, such as *M. mulatta*, have a clearer distinction between submission (SBT) and friendliness (lip-smacking), this distinction is blurred in more tolerant species, like *M. tonkeana* or *M. nigra*. For instance, Clark et al. (2020) were able to identify morphological subtypes of the SBT display in *M. nigra* linked to distinct contexts ranging from affiliation over play and copulation to submission. While it is unclear whether such morphological subtypes also exist in *M. mulatta*, context-defined functional subtypes of SBTs have been reported: Conflict SBTs (cSBTs) are shown as signs of immediate submission in aggressive encounters, whereas peaceful SBTs (pSBTs) are preemptively presented as signs of formal hierarchical subordination (Beisner and McCowan 2014), as previously observed in *M. nemestrina* (Flack and de Waal 2007). We hypothesize that some SBT displays in our study were interpreted as submission or fear in order to avoid an imminent threat (cSBTs), leading to the choice of the SBT target (or confusion with the Threat target), whereas other SBTs were perceived as more tolerant, appeasing or affiliative (pSBTs), entailing confusion with the lip-smacking category. This interpretation may also explain the inconsistent evaluation of SBTs reported by Taubert and Japee (2024). The basis for distinguishing these two SBT subtypes could include subtle facial signs of tension, specific combinations of AUs or AU intensities, or the factors identified by our MLR analysis, body weight and gaze aversion. High body weight of the stimulus monkey reduced the likelihood for the selection of the SBT target, suggesting that a facial display by a large, presumably strong monkey was unlikely to be perceived as submission. Direct gaze appeared important for lip-smacking gestures to be categorized correctly, as gaze aversion decreased the probability for the selection of the Lipsmack target. Conversely, gaze aversion (potentially signaling avoidance) increased the chances of an expression to be sorted into the avoidance-congruent SBT category. Averting gaze during a lip-smacking gesture might create the impression of underlying fear and/or avoidance, contradicting the approach-indicating mouth display, thereby leading with higher probability to the choice of the avoidance-associated SBT category. This aligns with studies in humans reporting a facilitated recognition of approach-associated expressions (happiness and anger) with direct gaze and a facilitated recognition of avoidance-associated expressions (fear and sadness) with averted gaze due to congruent behavioral intent signaled by gaze and facial expression (Adams and Kleck 2003, 2005). These findings collectively highlight the nuance of macaque facial behavior and reveal limits of mouth movement-defined expression categories, calling for further research into which variations in facial displays carry communicative meaning.

### Mouth, eyebrow and ear movements were integrated to discriminate facial expressions

In part II, we exploited a fully controllable monkey avatar to map out which facial motion and shape features are important for the perception and differentiation of the four facial expression categories. The two macaques’ categorization frequencies of expressions morphed between two prototypical expressions closely followed the choice frequencies to be expected according to the intensity of each expression category in the respective video. Moreover, increasing threat intensity was paralleled by increasing pupil size. This suggests that expressions were perceived along a continuum beyond simple template matching. This graded sensitivity may reflect the macaques’ ability to extract multifaceted information from natural facial displays, as behavioral contexts require assessment of expression strength, e.g. distinguishing mild threat from severe aggression, or evaluation of blended signals, e.g. appeasement displays despite aggressive impulses or threat displays despite fearful disposition. Random forest classification using the automatically quantified composite FACS predictors showed that the degree of jaw opening, teeth baring and lip puckering expectedly predicted the choice probabilities of the Threat, SBT and Lipsmack categories, respectively, and revealed that lowered brows in ambiguous expressions often informed the choice of the SBT instead of the Threat target, whereas erected ears served as a predictor for the choice of the Threat target. Under the assumption that the SBT category in our study was predominantly interpreted as a submissive signal to avoid conflict (cSBTs) (Beisner and McCowan 2014; Flack and de Waal 2007), lowering the brows and thus protecting the eye region against an imminent threat would be an adaptive behavior (Andrew 1963). Conversely, raising of the brows during SBTs given as signs of formal subordination (pSBTs) could serve to enlarge the visual field for attentive monitoring of more distant possible threats, as has been implicated for human wide-eyed fear expressions (Susskind et al. 2008). However, given the restricted nature of our stimulus set and the small sample size of two monkeys, these conclusions should be taken with caution and used as the basis for further research explicitly probing and manipulating the role of eyebrow and ear movements in facial displays, which are currently underexplored in the literature. Anecdotal accounts of eyebrow movements in macaques describe lowered eyebrows combined with a staring gaze, as well as “eyebrow-flashes” (brief raising of the eyebrows) as low-grade threats, unless combined with lip-smacking (Fortman et al. 2001; Wolfensohn and Lloyd 2003). Rincon et al. (2023) examined the specificity of FACS AU use in rhesus macaques across different contexts and found that they raised their brows mostly in aggressive contexts and less so in submissive or affiliative situations, whereas lowered eyebrows were almost exclusively displayed in submissive contexts. Erected ears (EAU1 or EAU2) occurred in aggressive encounters, whereas flattened ears were registered in all three contexts. In a similar vein, Partan (2002) reported that pant-threat vocalizations were most frequently accompanied by forward facing ears. Humans possess little ear muscle control and thus ear movements do not play a role in facial expressions, whereas human eyebrow movements are extensive and nuanced, but do not seem diagnostic for a specific facial expression category (Mielke et al. 2022). It has been suggested that eyebrows are moved according to the momentary appropriate biological function of increasing or decreasing the visual input (Ekman 1979, 2004; Susskind et al. 2008).

### Expression recognition transcended motion coherence and appearance degradation but required knowledge of social meaning

We found that temporal continuity of motion was not necessary for the perception of a facial expression, as frame-scrambled prototypical avatar expressions were well recognized by the two tested macaques and elicited the same pupil reactions as temporally coherent sequences. Furthermore, fully naturalistic facial appearance was not critical, as the prototypical monkey expressions displayed by furless and colorless monkey avatars were still largely correctly categorized and accompanied by differential pupil sizes. The furless and colorless avatars were previously shown to be perceived as “uncanny”, causing gaze aversion and distressed facial reactions (Siebert et al. 2020). The current findings of preserved categorization and pupil size modulation, underline that these avatars were realistic enough to be perceived as biologically plausible agents, which tend to evoke feelings of eeriness towards realistic, yet not fully realistic artificial agents (“uncanny valley”, Mori, 1970/2012). This becomes clearest in contrast to the even more unrealistic bare wireframe head avatar that did not elicit aversion in Siebert et al. (2020) and for which categorization failed in the present study, mirrored by a lack of pupil size differences between expressions. We found the same results for form-from-motion point-light expressions. This indicates that the tested macaques required discernable facial shape and somewhat realistic texture to interpret facial movements as meaningful facial expressions. Point-light stimuli are widely used in human research (for a review see e.g. Giese and Poggio (2003), but behavioral discrimination studies using point-light stimuli in macaques are rare and discrimination based on such stimuli may require training (Vangeneugden et al. 2010).

When monkey facial expressions were displayed by a human avatar, the tested macaques did not categorize them well. Previous findings with human observers showed that expressions were recognized irrespective of whether they were displayed by a human avatar or a monkey avatar (Taubert et al. 2021), aligning with the hypothesis that facial dynamics are represented independently of facial form (Bernstein et al. 2018; Bernstein and Yovel 2015; Duchaine and Yovel 2015), which could be achieved via norm-referenced encoding of facial expression in terms of difference to a neutral (norm) face within a particular shape domain (Stettler et al. 2026). Our data from two animals suggests that the recognition of monkey facial expressions may not be similarly invariant to species-specific facial shape in rhesus macaques, however, more research is needed.

Human facial expressions received unsystematic categorizations and did not elicit differential pupil reactions, suggesting that they were not mapped onto the trained categories by the one macaque who could be tested with human expressions. This argues against a homologous interpretation of human and macaque expressions. Categorizations followed neither presumed homologous intent (smiling/happy and lip-smacking, gasping/fearful and SBT, scowling/angry and threat), nor proposed phylogenetic homology, such as human smiles and macaque SBT displays (e.g. Kanazawa (1996), but compare Preuschoft (1992), Preuschoft and van Hooff (1995) and van Hooff (1967) for discussions on phylogenetic homology in facial displays). Moreover, the categorization pattern was not indicative of low-level feature matching, as human expressions sharing elements with monkey expressions, such as tooth exposure and retracted lip corners in smiles and SBT displays, or mouth opening in gasping (fear) expressions and threats, were not categorized accordingly. Instead, FACS-based visual similarities between human expressions and monkey expressions were uncorrelated with the target choice frequencies. This failure to categorize human expressions likely reflects limited experience, as monkeys in our facility rarely see human faces without masks, let alone have the opportunity to learn behavioral consequences associated with distinct human facial expressions. Without learned associations linking these facial configurations to behavioral intentions and predicted outcomes, human expressions remained socially meaningless to the tested macaque, eliciting neither systematic categorization nor differential arousal. These results accord with Zhu et al. (2013), who found no differential fMRI activations for human facial expressions in macaques and Sugase et al. (1999) who recorded markedly decreased neural response magnitudes for human compared to monkey facial expressions. While some studies have used human facial expressions to probe visual encoding mechanisms of subtle facial feature changes in the macaque brain (e.g. Wehrheim et al. 2026), our findings highlight the importance of using conspecific facial expressions for connecting visual perception to social information.

Thus, we conclude that facial expression perception in the present study emerged from integrating expression-characteristic visual features with social knowledge, enabling flexible, context-sensitive interpretation of communicative intent.

### Limitations

The main limitation of our study is the small sample size of only two subjects, constraining the scope of our conclusions to the tested individuals, and precluding averaging of data at the stimulus level (except for the binomial test), deployment of repeated measures analyses, or inclusion of “Subject” as a random factor in the MLR/GLM analyses. As a consequence, the reported analyses derive statistical power from repeated measurements per subject and condition rather than from independent replicates across individuals. Therefore, the reported degrees of freedom and test statistics reflect the number of trials rather than the number of biological samples, and should not be read as indicating effect magnitude or generalizability to the species. However, the converging pattern of results across the two subjects suggests a common underlying mechanism, calling for future systematic investigations in a larger number of rhesus macaques.

## Supplementary Information

Below is the link to the electronic supplementary material.

Supplementary Material 1

## Data Availability

All data are available on the Open Science Framework repository at https://osf.io/y3gc8/?view_only=f17d5149e32e4a94ba3d0940b6f32b3f.
