# Supplementary material for: Behavioral characterization of dynamic facial expression perception in rhesus monkeys (Macaca mulatta) using naturalistic and synthetic stimuli
Source: Anim Cogn. 2026 Oct 5;29(1):71. doi: 10.1007/s10071-026-02109-6 (PMC13672597; doi:10.1007/s10071-026-02109-6)
Supplement: Supplementary file 1 [file 10071_2026_2109_MOESM1_ESM.docx]

**SUPPLEMENTARY**

**Methods: Generation of avatars**

The creation of the dynamic monkey head avatar is described in detail elsewhere (Siebert et al., 2020; Taubert et al., 2021). In short, the monkey head model was based on MRI scans transformed into a mesh surface, which was controlled by embedded ribbons modeled after the macaque's facial muscles (Parr, Waller, Burrows, Gothard, & Vick, 2010). The ribbon control joints corresponded to motion-captured infrared markers, which had been attached to a monkey’s face in order to record the facial movements with a VICON FX20 Motion Capture System. Human facial movements were recorded analogously from a 40-year-old male human subject. The segmented movement trajectories were preprocessed with VICON Nexus 1.85 software and time-normalized and smoothed with a generative Bayesian method combining Gaussian process latent variable models and Gaussian process dynamic models, which allows for morphing between expressions (see Taubert et al. (2021) Appendix 1 for details on the algorithm). The textures and skin material of the monkey avatar were hand-painted using photographic references, and additional texture maps were created in Adobe Photoshop to represent various skin layers. The fur was simulated with Autodesk Maya’s XGen Interactive Grooming tool. All movie frames were generated with Autodesk Arnold Renderer. The human avatar was based on a female face scan and texture maps provided by EISKO, with custom adjustments to the color and texture maps to match the monkey head model. The movement deformations were achieved via control points equivalent to the monkey motion-captured markers, following the human muscle anatomy (Taubert et al., 2021).

This resulted in a monkey head avatar able to display the prototypical, naturalistic neutral, lip-smacking, SBT and threat expressions, as well as expressions morphed across two categories in steps of 25 % intensity between each pair of prototypical expressions. Additionally, we tested scrambled-frame versions of the prototypical expressions (lacking temporal continuity of the movement), dynamic point-light expressions, which contained the same movement patterns as the prototypical naturalistic avatars, but no texture (instead white dots of 200 ms limited-lifetime distributed over the avatar surface mesh on a black background), and less realistic render types of the monkey avatar, which had been used in a previous study to test for an “uncanny valley” of aversion towards artificial agents (Siebert et al., 2020): an unnatural wireframe head without texture; a textured, but grayscale avatar; and a colored, but still furless avatar. Furthermore, we tested the monkeys’ categorization of the human avatar displaying monkey expressions, the human avatar presenting human expressions (smiling/happy, gasping/fearful, scowling/angry) and the monkey avatar showing those human expressions.

**Supplementary Tables and Figures**

**Table S1** Overview of automatic MaqFACS (Macaque Facial Action Coding System) AU (Action Unit) activation and intensity calculation. Note that AU6 was not quantified, because it was not present in our stimuli and because appropriate markers are not available in the DLC primate face model

| **Action Unit (AU)** | **Action Unit Descriptor** | **Intensity calculation description** |
| --- | --- | --- |
| AU1_2 | brow raiser | max. distance of Brow_y (average of BrowTop_R_y, Brow_Mid_y, BrowTop_L_y) upwards relative to its start position |
| AU41 | glabella lowerer | max. distance of Brow_y downwards relative to its start position |
| AU8 | lips toward each other | abs. min. distance between LipUpper_Mid_y and LipLower_Mid_y relative to their start distance |
| AU9 | nose wrinkle | max. distance of Nose_Mid_y upwards relative to its start position |
| AU10 | upper lip raiser | max. distance of LipUpper_Mid_y upwards relative to its start position |
| AU12 | lip corner puller | max. distance between LipCorner_R and LipCorner_L larger than their start distance |
| AU16 | lower lip depressor | abs. min. distance between LipUpper_Mid_y and Chin_Mid_y smaller than their start distance |
| AU18i | true pucker | abs. min. distance between LipCorner_R_x and LipCorner_L_x smaller than their start distance. If AU25 larger than 3 pixels was present throughout the presence of AU18, AU18ii (outer pucker) was scored instead (however not present in our stimuli) |
| AU25 | lips parted | max. distance between LipUpper_Mid_y and LipLower_Mid_y relative to their start distance |
| AU26/AU27 | jaw drop/ mouth stretch | max. distance of Chin_Mid_y downwards relative to its start position; AU26 and AU27 were not distinguishable automatically and were thus pooled |
| EAU1 | ears forward | max. distance between Ear_R_x and Ear_L_x relative to their start distance |
| EAU2 | ear elevator | max. distance of Ears_y (average trace of Ear_L_ y and Ear_R_y) upwards relative to its start position |
| EAU3 | ear flattener | abs. min. distance between Ear_R_x and Ear_L_x relative to their start distance |
| AD181 (Action Descriptor) | lip-smacking | the number of local minima in the position of LipUpper_Mid_y, when at least one local minimum and at least one local maximum, each with min. peak prominence 1, were present |

**Fig. S1** Categorization performances for the training stimuli. **(A)** Performance of monkey 1 for each expression category during the final training phase, in which trials were presented fully randomized. **(B)** Performance of monkey 2 during the final training phase. **(C)** Performance of monkey 1 on the trained videos for each test session. The left panel shows the main test phase, the right panel shows the later test phase, during which only the human expressions were tested. **(D)** Performance of monkey 2 on the trained videos for each test session

**Fig. S2** **(A)** Visual similarity matrices per test ID for all pure test expressions and visual similarity matrices between human expressions and monkey expressions. Visual similarity was calculated as the Pearson correlation between the quantified FACS compositions of an expression pair. **(B)** Difference matrices per test ID for all pure test expressions, for monkey 1 (M1) and monkey 2 (M2) separately. Differences were calculated between observed relative choice frequency and the expected choice frequency according to the visual similarity between the test expression and the expression associated with the selected target. A value of zero (white) indicates that the target was selected as often as predicted by the visual similarity, positive values (red) indicate that the target was selected more often, and negative values (blue) indicate that the target was selected less often

**Fig. S3** Boxplots of pupil sizes during the viewing of each expression category of each stimulus identity for monkey 1 (M1) and monkey 2 (M2). Systematic pupil size differences between stimulus identities are influenced by differences in stimulus type brightness. **(A)** New monkey identities (ID1-4), naturalistic monkey avatar, and previously seen monkey identities with previously unseen expression categories (Training IDs/New expr.), complete expression repertoire (ANOVA Expression x Identity, post-hoc Tukey-Kramer tests). Note that the Training IDs/New expr. trials were not included in the ANOVA in order to enable post-hoc comparisons between expressions per stimulus identity, as each expression was displayed by a different identity for this stimulus set. Therefore, no indications of significant differences between expression categories of the Training IDs/New expr. can be provided. **(B)** New monkey identities with incomplete expression repertoire, SBT missing (ID 5-9), and average pupil size for these identities per expression, right-most panel (ANOVA Expression x Identity, post-hoc Tukey-Kramer tests)

**Table S2** Multinomial Logistic Regression (MLR) model comparisons for categorization choices of the pure (real and naturalistic monkey avatar) expression videos for monkey 1 (M1, *N* = 2012 trials)/monkey 2 (M2, *N* = 2074 trials)

| **MLR Model Nr.** | **Predictors [number of predictors]** | ***Adj. R^2^* (explained variance) (M1/M2)** | **Deviance *D* (from a perfect model) (M1/M2)** | **Δ Deviance *D*, *p* (M1/M2)** | ***Chi^2^*, df, *p* (M1/M2)** |
| --- | --- | --- | --- | --- | --- |
| 1 | Expression [1] | 0.1237/ 0.2010 | 4642.69/ 4009.70 | – | 683.04/ 1038.48, 9, <0.0001 |
| 2 | Pupil size [1] | 0.0335/ 0.0986 | 5135.22/ 4538.53 | – | 190.51/ 509.65, 3, <0.0001 |
| 3 | Expression + Identity [2] | 0.2828/ 0.2953 | 3723.43/ 3461.40 | 919.26/ 548.29, <0.0001 (Δ to Model 1) | 1602.30/ 1586.78, 45, <0.0001 |
| 4 | FACS (LipPucker, TeethBare, JawOpen, BrowRaise, BrowLow, EarsErect, EarsFlat) [7] | 0.1567/ 0.1817 | 4443.02/ 4082.73 | – | 882.71/ 965.45, 21, <0.0001 |
| 5 | FACSx: FACS + 3-way interactions between mouth predictors (LipPucker, TeethBare, JawOpen) x brow predictors (BrowRaise, BrowLow) x ear predictors (EarsErect, EarsFlat) [19] | 0.2714/ 0.2498 | 3760.43/ 3667.38 | 682.59/ 415.35, <0.0001 (Δ to Model 4) | 1565.30/ 1380.80, 57, <0.0001 |
| 6 | FACS + Avert [20] | 0.2278/ 0.2254 | 4058.60/ 3856.12 | 384.43/ 226.61, <0.0001 (Δ to Model 4) | 1267.14/ 1192.06, 24, <0.0001 |
| 7 | FACS + Avert + Weight [21] | 0.4880/ 0.4485 | 2667.02/ 2724.01 | 1391.58/ 1132.10, <0.0001 (Δ to Model 5) | 2658.72/ 2324.17, 27, <0.0001 |

**Table S3** General Linear Model (GLM) model comparisons for pupil size when viewing the pure (real and naturalistic monkey avatar) expression videos for monkey 1 (M1, N = 2007 trials)/monkey 2 (M2, N = 2067 trials)

| **GLM Model Nr.** | **Predictors [number of predictors]** | ***Adj. R^2^* (explained variance) (M1/M2)** | **Deviance *D* (from a perfect model) (M1/M2)** | **Δ Deviance *D*, *p* (M1/M2)** | ***F*, df, df_error_, *p* (M1/M2)** |
| --- | --- | --- | --- | --- | --- |
| 1 | Brightness [1] | 0.3111/ 0.1625 | 737.31/ 1388.16 | – | 906.97/ 401.96, 1, 2005/ 2065, *p*<0.0001 |
| 2 | Brightness + Expression [2] | 0.3742/ 0.4379 | 668.82/ 930.32 | 68.49/ 457.84, <0.0001 (Δ to Model 1) | 300.84/ 403.42, 4, 2005/ 2062, *p*<0.0001 |
| 3 | Brightness + Expression + Identity [3] | 0.4047/ 0.5261 | 632.46/ 779.84 | 36.40/ 150.78, <0.0001 (Δ to Model 2) | 86.22/ 144.34, 16, 1990/ 2050, *p*<0.0001 |
| 4 | Brightness + FACS (LipPucker, TeethBare, JawOpen, BrowRaise, BrowLow, EarsErect, EarsFlat) [8] | 0.3800/ 0.4136 | 661.30/ 968.72 | 76.02/ 419.44, <0.0001 (Δ to Model 1) | 154.67/ 183.14, 8, 1998/ 2058, *p*<0.0001 |
| 5 | Brightness + FACSx: FACS + 3-way interactions between mouth predictors (LipPucker, TeethBare, JawOpen) x brow predictors (BrowRaise, BrowLow) x ear predictors (EarsErect, EarsFlat) [20] | 0.4242/ 0.4968 | 610.48/ 826.40 | 50.82/ 142.32, <0.0001 (Δ to Model 4) | 74.88/ 102.99, 20, 1986/ 2046, *p*<0.0001 |
| 6 | Brightness + FACS + Avert [21] | 0.3950/ 0.4420 | 644.95/ 921.26 | 34.46/ 94.86, <0.0001 (Δ to Model 5) | 146.53/ 182.87, 9, 1997/ 2057, *p*<0.0001 |
| 7 | Brightness + FACS + Avert + Weight [22] | 0.2929/ 0.4799 | 499.46/ 675.21 | 145.49/ 246.05, <0.0001 (Δ to Model 6) | 64.67/ 149.58, 10, 1520/ 1600, *p*<0.0001 |
| 8 | Brightness + Total AUs Intensity [2] | 0.3479/ 0.3108 | 679.96/ 1141.83 | 39.35/ 246.33, <0.0001 (Δ to Model 1) | 535.31/ 466.85, 2, 2004/ 2064, *p*<0.0001 |
| 9 | Brightness + Number of activated AUs [2] | 0.3357/ 0.2845 | 710.63/ 1185.33 | 26.69/ 202.83, <0.0001 (Δ to Model 1) | 507.91/ 411.85, 2, 2004/ 2064, *p*<0.0001 |

**Fig. S4** **(A)** Log odds coefficients for choosing each target relative to choosing the Neutral target (reference category) in MLR model 1 with expression category as predictor. **(B)** Log odds coefficients of MLR model 2 with pupil size as predictor. **(C)** Log odds coefficients of MLR model 7 with the FACS composition, gaze aversion (Avert) and body weight (Weight) as predictors. Error bars represent 95% confidence intervals

**Fig. S5 (A)** Relative choice frequencies of monkey 2 for all morphed avatar videos, shown for each morph axis between the prototypical expressions Neutral, Lipsmack, SBT and Threat separately, N per video = 18 trials. **(B)** Swarm plots show pupil sizes of monkey 1 (M1) and monkey 2 (M2) during the viewing of the morphed avatar expressions, separated by expression intensity and color-coded by morph axis. Averages for each morph axis are plotted as large symbols with SEM error bars and linear trend lines are overlaid

**Table S4** Multinomial Logistic Regression (MLR) model comparisons for categorization choices of the morphed avatar expressions for monkey 1 (M1, N = 1098 trials)/monkey 2 (M2, N = 334 trials)

| **Morphs MLR Model Nr.** | **Predictors [number of predictors]** | ***Adj. R^2^* (explained variance) (M1/M2)** | **Deviance *D* (from a perfect model) (M1/M2)** | **Δ Deviance *D*, *p* (M1/M2)** | ***Chi^2^*, df, *p* (M1/M2)** |
| --- | --- | --- | --- | --- | --- |
| 1 | Lipsmack, SBT and Threat intensities [3] | 0.1765/ 0.2050 | 2431.90/ 691.98 | – | 550.35/ 208.58, 9, <0.0001 |
| 2 | Lipsmack, SBT and Threat intensities + 2-way interactions [6] | 0.1798/ 0.1970 | 2404.10/ 681.15 | 27.80/ 10.83, <0.001/ 0.013 (Δ to Model 1) | 578.15/ 219.42, 18, <0.0001 |
| 3 | FACS (LipPucker, TeethBare, JawOpen, BrowRaise, BrowLow, EarsErect, EarsFlat) [7] | 0.1761/ 0.1867 | 2409.21/ 684.43 | – | 573.04/ 216.14, 21, <0.0001 |

**Table S5** General Linear Model (GLM) model comparisons for pupil size when viewing the morphed avatar expressions for monkey 1 (M1, N = 1097 trials)/monkey 2 (M2, N = 334 trials)

| **Morphs GLM Model Nr.** | **Predictors [number of predictors]** | ***Adj. R^2^* (explained variance) (M1/M2)** | **Deviance *D* (from a perfect model) (M1/M2)** | **Δ Deviance *D*, *p* (M1/M2)** | ***F*, df, df_error_, *p* (M1/M2)** |
| --- | --- | --- | --- | --- | --- |
| 1 | Brightness [1] | 0.0339/ 0.0069 | 462.54/ 209.77 | – | 39.40/ 3.33, 1, 1095/ 332, <0.0001/ 0.069 |
| 2 | Brightness + Lipsmack, SBT and Threat intensity [4] | 0.1115/ 0.0348 | 424.19/ 202.04 | 38.35/ 7.73, <0.0001/ 0.052 (Δ to Model 1) | 35.39/ 4.00, 4, 1092/ 329, <0.0001/ 0.0035 |
| 3 | Brightness + Lipsmack, SBT and Threat intensities + 2-way interactions [7] | 0.1115/ 0.0348 | 423.04/ 200.20 | 1.14/ 1.84, 0.77/ 0.61 (Δ to Model 2) | 20.64/ 2.71, 7, 1089/ 326, <0.0001/ 0.0095 |
| 4 | Brightness + FACS (LipPucker, TeethBare, JawOpen, BrowRaise, BrowLow, EarsErect, EarsFlat) [8] | 0.1139/ 0.0458 | 421.50/ 197.30 | 41.04/ 12.46, <0.0001/ 0.086 (Δ to Model 1) | 18.61/ 3.00, 8, 1088/ 325, <0.0001/ 0.0029 |
| 5 | Brightness + Total AUs Intensity [2] | 0.0361/ 0.0188 | 461.04/ 206.63 | 1.50/ 3.14, 0.22/ 0.076 (Δ to Model 1) | 21.53/ 4.20, 2, 1094/ 331, <0.0001/ 0.016 |
| 6 | Brightness + Number of activated AUs [2] | 0.0376/ 0.0189 | 460.40/ 206.62 | 2.20/ 3.14, 0.14/ 0.076 (Δ to Model 1) | 23.39/ 4.20, 2, 1094/ 331, <0.0001/ 0.016 |

**Fig. S6** Select models of choice and pupil size data in response to the morphed avatar expressions for monkey 2. All model details are available in Supplementary Table S4 (choice) and Supplementary Table S5 (pupil size). Error bars represent 95% confidence intervals. **(A)** Log odds coefficients for choosing each target relative to choosing the Neutral target (reference category) in Morphs MLR Model 1 with expression intensities in the morphed expressions as predictors. **(B)** Log odds coefficients in Morphs MLR Model 3 with the composite FACS predictors. **(C)** Beta coefficients in Morphs GLM Model 2 with expression component intensities in the morphed expressions and video brightness as predictors of the pupil size

**Fig. S7** Random Forest Classification results of the morphed avatar expressions using the full set of FACS AUs as predictors (full AU model). **(A)** Heatmap showing the predicted category vs. the chosen category. **(B)** Out-of-bag feature importance of the predictors. **(C)** Simplified decision tree

**Fig. S8** Random Forest Classification results of the pure (unambiguous) real and naturalistic avatar expressions using the composite FACS predictors (composite model). **(A)** Heatmap showing the predicted category vs. the chosen category. **(B)** Out-of-bag feature importance of the predictors. **(C)** Simplified decision tree. **(D)** Partial dependency plots

**Fig. S9** Random Forest Classification results of the morphed avatar expressions using the expression intensities in the morphs as predictors. **(A)** Heatmap showing the predicted category vs. the chosen category. **(B)** Out-of-bag feature importance of the predictors. **(C)** Simplified decision tree. **(D)** Partial dependency plots

**Fig. S10 (A)** Expression categorization frequencies of avatar variants for monkey 2, asterisks indicate correct categorization significantly above chance level (binomial tests, p < 0.05, FDR-corrected). The first four avatar types increase in realism: point-light expressions (N per video = 29 trials), wireframe mesh (N per video = 15 trials), grayscale avatar (N = 15 trials) and furless avatar (N per video = 15 trials). Additionally, a human avatar with monkey expressions (N per video = 29 trials), and a naturalistic monkey avatar with time-scrambled frame order (N per video = 30 trials) were tested. **(B)** Difference in fraction of correct categorizations of avatar variants relative to the (now trained) naturalistic avatar per expression category for monkey 2. Percentages at the bottom indicate the overall difference in correct categorizations for each avatar type. **(C)** Boxplots of pupil sizes of monkey 2 during the viewing of each expression category for all avatar variants. ANOVA: Expression F(2, 459) = 4.37, p = 0.013, AvatarType F(4, 459) = 311.57, p < 0.001, Expression x AvatarType F(14, 459) = 3.18, p < 0.001) ; *p < 0.05, post-hoc Tukey-Kramer tests. The difference in pupil size between the Neutral and the Threat category for the Gray Avatar marginally missed significance at p = 0.071. Systematic pupil size differences between avatar types are influenced by differences in stimulus type brightness

**References**

Parr, L. A., Waller, B. M., Burrows, A. M., Gothard, K. M., & Vick, S. J. (2010). MaqFACS: A muscle-based facial movement coding system for the rhesus macaque. *American Journal of Physical Anthropology, 143*(4), 625-630. doi:10.1002/ajpa.21401

Siebert, R., Taubert, N., Spadacenta, S., Dicke, P. W., Giese, M. A., & Thier, P. (2020). A Naturalistic Dynamic Monkey Head Avatar Elicits Species-Typical Reactions and Overcomes the Uncanny Valley. *eNeuro, 7*(4). doi:10.1523/ENEURO.0524-19.2020

Taubert, N., Stettler, M., Siebert, R., Spadacenta, S., Sting, L., Dicke, P., . . . Giese, M. A. (2021). Shape-invariant encoding of dynamic primate facial expressions in human perception. *Elife, 10*. doi:10.7554/eLife.61197
